# A hydrophobic residue stabilizes dimers of regulatory ACT-like domains in plant basic helix–loop–helix transcription factors

**DOI:** 10.1016/j.jbc.2021.100708

**Published:** 2021-04-24

**Authors:** Yun Sun Lee, Andres Herrera-Tequia, Jagannath Silwal, James H. Geiger, Erich Grotewold

**Affiliations:** 1 Department of Biochemistry & Molecular Biology, Michigan State University, East Lansing, Michigan, USA; 2 Department of Chemistry, Michigan State University, East Lansing, Michigan, USA

**Keywords:** anthocyanins, yeast two-hybrid, protein–protein interaction, R, GL3, ACT, *a*spartate kinase, *c*horismate mutase, and *T*yrA, ALPHA, amplified luminescent proximity homogeneous assay, 3-AT, 3-amino-1,2,4-triazole, bHLH, basic helix–loop–helix, GL3^ACT^, ACT-like domains of GL3, GST, glutathione-*S*-transferase, pAD, GAL4 activation domain, pBD, GAL4 DNA-binding domain, PDB, Protein Data Bank, PheH, phenylalanine hydroxylase, PPI, protein–protein interaction, R^ACT^, ACT-like domains of R, SD, synthetically defined, TCEP, Tris(2-carboxyethyl)phosphine, TF, transcription factor, Y2H, yeast-two hybrid

## Abstract

About a third of the plant basic helix–loop–helix (bHLH) transcription factors harbor a C-terminal *a*spartate kinase, *c*horismate mutase, and *T*yrA (ACT)-like domain, which was originally identified in the maize R regulator of anthocyanin biosynthesis, where it modulates the ability of the bHLH to dimerize and bind DNA. Characterization of other bHLH ACT-like domains, such as the one in the *Arabidopsis* R ortholog, GL3, has not definitively confirmed dimerization, raising the question of the overall role of this potential regulatory domain. To learn more, we compared the dimerization of the ACT-like domains of R (R^ACT^) and GL3 (GL3^ACT^). We show that R^ACT^ dimerizes with a dissociation constant around 100 nM, over an order of magnitude stronger than GL3^ACT^. Structural predictions combined with mutational analyses demonstrated that V568, located in a hydrophobic pocket in R^ACT^, is important: when mutated to the Ser residue present in GL3^ACT^, dimerization affinity dropped by almost an order of magnitude. The converse S595V mutation in GL3^ACT^ significantly increased the dimerization strength. We cloned and assayed dimerization for all identified maize ACT-like domains and determined that 12 of 42 formed heterodimers in yeast two-hybrid assays, irrespective of whether they harbored V568, which was often replaced by other aliphatic amino acids. Moreover, we determined that the presence of polar residues at that position occurs only in a small subset of anthocyanin regulators. The combined results provide new insights into possibly regulatory mechanisms and suggest that many of the other plant ACT-like domains associate to modulate fundamental cellular processes.

Control of gene expression relies on the proper organization of transcription factors (TFs) and other proteins on gene regulatory regions. This is largely accomplished through protein–DNA and protein–protein interactions (PPIs). Thus, in addition to harboring DNA-binding domains that interpret the *cis*-regulatory code in the genome, TFs are characterized by the presence of one or more PPI domains. The same TF can regulate different sets of genes in different cell types or conditions, a consequence of their ability to form different complexes as part of what is known as combinatorial control (1). Thus, determining how PPI domains participate in TF assembly is fundamental to understand gene regulation.

The basic helix–loop–helix (bHLH) family of TFs is among the largest in animals (2) and plants (3). The bHLH domain is structurally conserved, and it is about 60 amino acids long organized into two functionally distinct regions (4, 5). The N-terminal basic region is responsible for binding to the canonical E-box DNA motif (CANNTG), but DNA recognition requires the formation of homodimers or heterodimers with other bHLH proteins, and such PPIs are mediated by the HLH region (6). In addition to the bHLH domain, members of this TF family often harbor other conserved motifs that participate in PPIs and that have contributed to the classification of the family (3, 7, 8). Some of these motifs are shared between plant and animal bHLH TFs, such as the leucine zipper motif that is often present immediately C terminal to the second helix of the HLH motif, and are important in stabilizing bHLH-mediated dimer formation and providing DNA-binding specificity to the homodimers or heterodimers (9, 10, 11). Other motifs appear to be specific to the plant kingdom and include the ACT-like domain, a fold first identified in the aspartate kinase, chorismate mutase, and TyrA enzymes (hence the name, ACT) (12) and later shown to be present in about a third of the plant bHLH TFs (13). Similar to the structurally related bHLH protein interaction and function domain (14, 15), the ACT-like domain can mediate PPIs (13, 16).

ACT domains are 70 to 80 amino acids long and have primarily been found in proteins involved in the regulation or biosynthesis of amino acids. When part of enzymes, they can participate in allosteric regulation by pathway intermediates, frequently involving the formation of homodimers, or higher order structures (17, 18). Several ACT structures have been solved, some bound to ligands (18), and those ACT domains that are part of enzymes generally show a βαββαβ topology (19), although flexibility in the structure of the domain is becoming evident as more structures are solved (20, 21, 22).

Maize R was the first plant bHLH regulator identified (23), and its function is to regulate the accumulation of anthocyanin pigments, by physically interacting with the R2R3–MYB domain (MYB domain harboring two MYB repeats most similar to the second and third MYB repeats of the product of the *c-myb* proto-oncogene) of C1 (24, 25). R homodimer formation and DNA binding require an extended bHLH that includes a short leucine zipper motif but is inhibited by dimerization of the C-terminal ACT-like domain, which has a ββαββα organization (26). Thus, the R ACT-like domain functions as a regulatory switch that dictates whether R-containing regulatory complexes are tethered to target genes through the bHLH or through the R2R3–MYB partner (26). R is a member of subgroup IIIf of plant bHLH proteins (3, 7). This subgroup also includes the partially redundant *Arabidopsis* GL3 (GLABRA3) and EGL3 (ENHANCER OF GL3) bHLH proteins, which participate in the specification of root epidermal cell fate (27, 28), in the control of trichome (leaf hair) formation (29, 30, 31), and in the control of anthocyanin accumulation (32), by interacting with different R2R3–MYB proteins, participating in the formation of different MYB, bHLH, and WD (tryptophan–aspartic acid repeat-containing proteins) complexes (33, 34). Notwithstanding the very similar domain structure of GL3/EGL3 and R, and the presence of an ACT-like domain (Fig. 1*A*), results are inconsistent on whether the C-terminal region of GL3 is capable of forming homodimers (35, 36).

**Figure 1:**
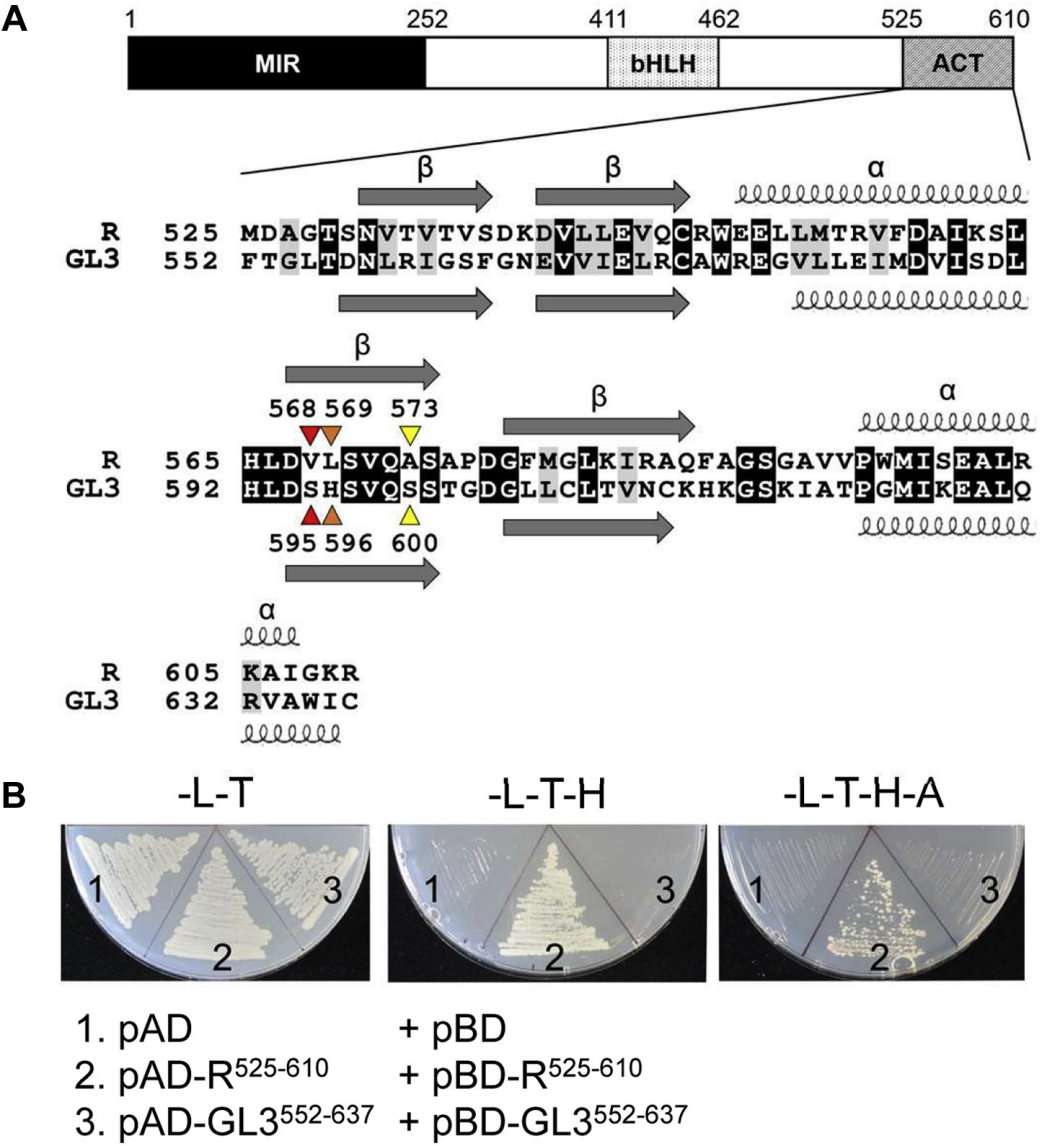
**Homodimerization of the R and GL3 ACT-like domains.***A*, amino acid alignment between the ACT-like domains of R (residues 525–610) and GL3 (residues 552–637). The alignment was generated using ClustalW in BioEdit, version 7 (60). Secondary structures were predicted from the ACT domain of phenylalanine hydroxylase (Protein Data Bank: 5FII). The resultant α-helices and β-strands are indicated by *spirals* and *horizontal arrows*, respectively. The numbers over *colored arrowheads* represent the amino acids that were substituted in the mutation assays, where the residues marked in same color were switched with each other. *B*, yeast two-hybrid assays probing interaction of R^525–610^ and GL3^552–637^ fused to the GAL4 activation domain (pAD) or GAL4 DNA-binding domain (pBD) in the yeast strain PJ69.4a (37) containing the *HIS3* and *ADE2* genes under the control of GAL4-binding sites. Interaction is manifested by growth on SD media deficient in Leu/Trp/His and Leu/Trp/His/Ade (–L–T–H, –L–T–H–A) for 5 days, whereas SD media–deficient Leu/Trp (–L–T) selects for the bait and pray plasmids. Three independent transformants were analyzed for each plasmid combination corresponding to three biological replicates. ACT, *a*spartate kinase, *c*horismate mutase, and *T*yrA; SD, synthetically defined.

Here, using a combination of yeast two-hybrid (Y2H) assays with the amplified luminescent proximity homogeneous assay (ALPHA), we demonstrate that similar to R, the ACT-like domain of GL3 can homodimerize, yet with significantly lower affinity. We used structural predictions combined with the limited sequence homology between these ACT-like domains to identify a key residue (Val 568 in R) that, when replaced with the corresponding residue in GL3 (Ser), significantly impairs dimerization strength. Conversely, the replacement of this Ser residue with Val in GL3 is sufficient to enhance dimerization by more than fivefold. The analysis of the distribution of these two residues across plant ACT-like domains showed that GL3 is likely the exception, since most ACT-harboring bHLH factors contain Val or other aliphatic residues at the equivalent 568 position. The evaluation of apparent equilibrium dissociation constants (*K*_*d*_) with ALPHA for the various wildtype and mutant ACT-like domains allowed us to correlate strength of interaction with Y2H assay results, providing insights regarding the meaning of Y2H results as well as a possible explanation for prior inconsistencies (35, 36). Taken together, our studies provide significant insights regarding the dimerization of ACT-like domains.

## Results

### The GL3 and R ACT-like domains dimerize with very different affinities

GL3 has a very similar domain organization as R, including a C-terminal region (residues 552–637) that shows the ββαββα secondary structure that characterizes the R ACT-like domain (R^ACT^, residues 525–610; Fig. 1*A*). We used the Y2H assay to investigate dimerization of GL3^552–637^, in conditions in which R^525–610^ shows robust dimerization (13). For this, we fused GL3^552–637^ to the GAL4 activation domain (in plasmid pAD–GL3^552–637^; harboring the *LEU2* selectable marker) and to the GAL4 DNA-binding domain (in plasmid pBD–GL3^552–637^; harboring the *TRP1* selectable marker). We transformed pAD–GL3^552–637^ and pBD–GL3^552–637^ into the PJ69.4 yeast strain (37) and assayed growth in synthetically defined (SD) media lacking leucine and tryptophan (–L–T); leucine, tryptophan, and histidine (–L–T–H); and leucine, tryptophan, histidine, and adenine (–L–T–H–A). In contrast to cells harboring pAD–R^525–610^ and pBD–R^525–610^, which grew robustly in –L–T–H–A media, no growth in –L–T–H (usually considered adequate for weak interactions) or –L–T–H–A (usually considered adequate for strong interactions) was observed for cells with pAD–GL3^552–637^ and pBD–GL3^552–637^ (Fig. 1*B*), which is consistent with published results (35). No growth in –L–T–H or –L–T–H–A media was observed for either R^525–610^ or GL3^552–637^ fused to pAD or pBD, in the presence of the corresponding empty plasmids (Fig. S1). The growth of the various yeast strains in selective media as an indication of PPI was complemented by β-galactosidase assays, taking advantage that the PJ69.4 yeast strain harbors the *LacZ* gene under a GAL4-controlled promoter (37). β-Galactosidase assays confirmed the results obtained in selective media (Fig. S2). We also compared the growth of the various strains shown in Figure S3 in –L–T–H media supplemented with increasing concentrations (10–50 mM) of the HIS3 enzyme inhibitor, 3-amino-1,2,4-triazole (3-AT). The results show almost a perfect correlation between the ability of cells to grow in the presence of 3-AT and the enhanced selection provided by omitting adenine from the media (Fig. S3, compare with Fig. S2).

Western blots of extracts of yeast cells expressing several proteins fused to the GAL4–AD and showing distinct interaction strengths in yeast showed similar levels of accumulation when using commercial antibodies to GAL4–AD (Fig. S4). These results indicate that the different strengths of interaction observed in yeast are unlikely a consequence of different stability of the proteins.

**Figure 2:**
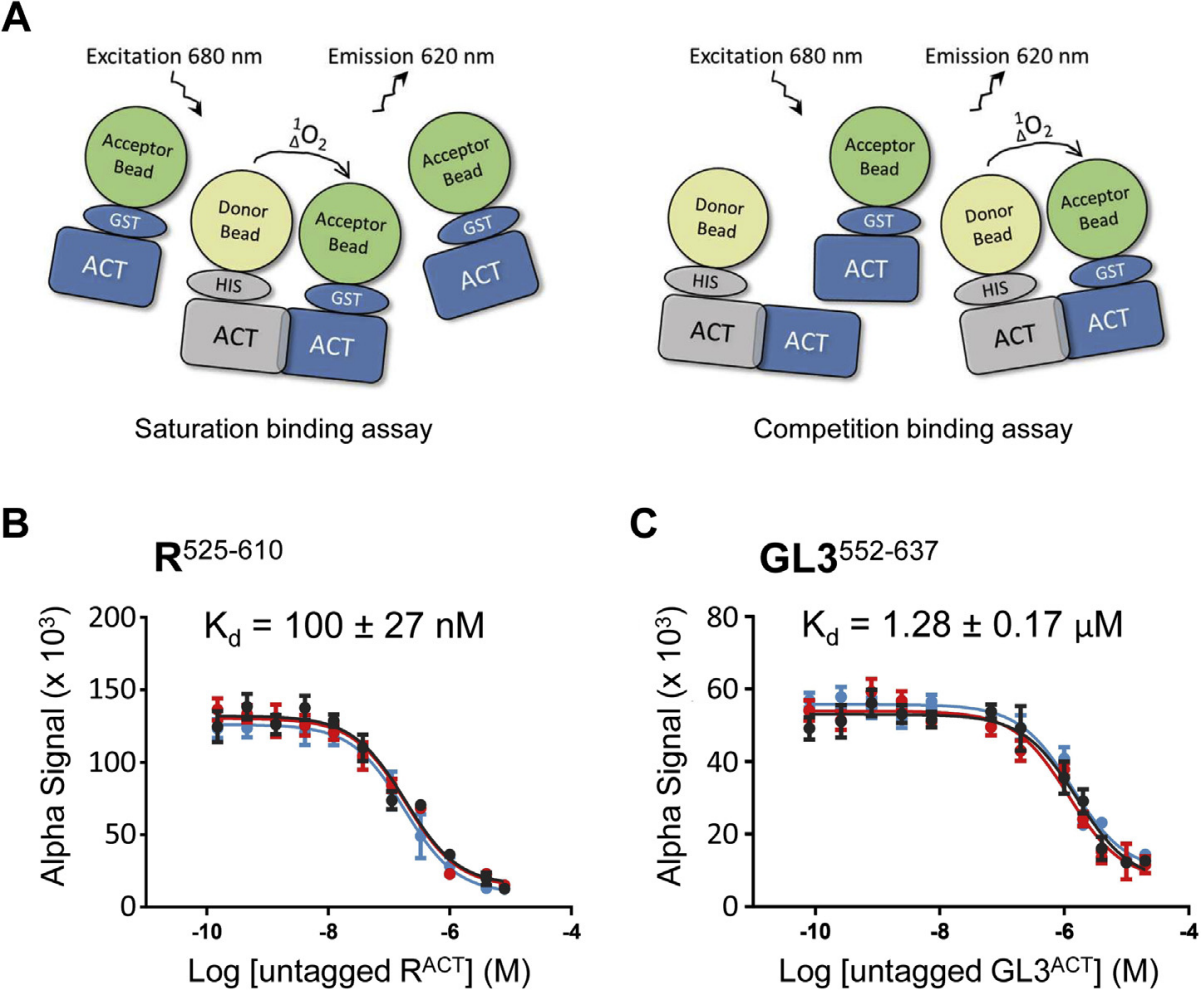
**Different binding strengths characterize the ACT-like domains of R and GL3.***A*, application of amplified luminescent proximity homogeneous assay (ALPHA) to determine dissociation constants (*K*_*d*_) of different combinations of ACT-like domains. When N_6_His-tagged and GST-tagged ACT-like domains interact, this brings in close proximity (<200 nm) the donor and acceptor beads, resulting in high-energy emission at 620 nm (61). Apparent *K*_*d*_ values are determined either by saturation (*left*) using excess of one protein over the other, or competition (*right*), using proteins that lack the N_6_His or GST tags. *B*, determination of the apparent *K*_*d*_ for R^ACT^ and *C*, GL3^ACT^ by competitive binding assays using ALPHA. Various concentrations of untagged R^ACT^ (0–8 μM) and GL3^ACT^ (0–10 μM) were incubated with a mixture of 100 nM of N_6_His-R^ACT^ and N_6_His-GL3^ACT^ and 100 nM of GST-R^ACT^ and GST-GL3^ACT^, respectively. The *K*_*d*_ values were calculated by one-site fit model. Each of the lines corresponds to one biological replicate, and each experiment was done in triplicate. The *K*_*d*_ values shown correspond to the average ± standard deviation. ACT, *a*spartate kinase, *c*horismate mutase, and *T*yrA; GST, glutathione-*S*-transferase.

To compare the dimerization binding affinities of GL3^552–637^ and R^525–610^, we expressed and affinity purified each protein fused to glutathione-*S*-transferase (GST) or the N_6_His tag (Fig. S5, *A*–*D*) and determined the respective *K*_*d*_ values using the ALPHA, by performing competition and saturation binding assays (Fig. 2*A*). For the competition binding assay, purified untagged (*i.e.,* without N_6_His or GST tag) versions of R^525–610^ and GL3^552–637^ (Fig. S5*E*) were used as competitors (Fig. 2*A* and Table 1). In agreement with R^525–610^ dimerizing very robustly (26), it showed a *K*_*d*_ of 100 nM (Fig. 2*B* and Table 1). In contrast, in identical conditions, GL3^552–637^ dimerization is characterized by a much higher *K*_*d*_ of 1.3 to 1.5 μM (Fig. 2*C* and Table 1). Based on these results, we conclude that GL3^552–637^ is capable of forming homodimers, but this interaction is too weak to be detected by Y2H.

**Table 1:** Summary of the dimerization strength of the ACT domains of R and GL3 evaluated by saturation binding using ALPHA and Y2H assays

Protein	Mutation	Y2H	Apparent *K*_*d*_ value (μM)^a^
R	WT	–L–T–H–A	0.10 ± 0.01
	V568S	No growth	1.72 ± 0.20
	L569H	–L–T–H–A	0.16 ± 0.03
	A573S	No growth	0.43 ± 0.06
	V568S/L569H	No growth	1.59 ± 0.23
	V568S/A573S	No growth	0.99 ± 0.17
	L569H/A573S	–L–T–H	0.11 ± 0.02
	V568S/L569H/A573S	No growth	1.51 ± 0.10
GL3	WT	No growth	1.54 ± 0.12
	S595V	–L–T–H	0.26 ± 0.01
	H596L	No growth	1.49 ± 0.24
	S600A	No growth	1.59 ± 0.52
	S595V/H596L	–L–T–H	0.27 ± 0.05
	S595V/S600A	–L–T–H	0.36 ± 0.04
	H596L/S600A	No growth	2.23 ± 0.33
	S595V/H596L/S600A	No growth	1.77 ± 0.19

a The *K*_*d*_ values represent the average ± standard deviation of three biological replicates.

### Identification of key dimer interface residues that specify interaction strength of ACT domains

**Figure 3:**
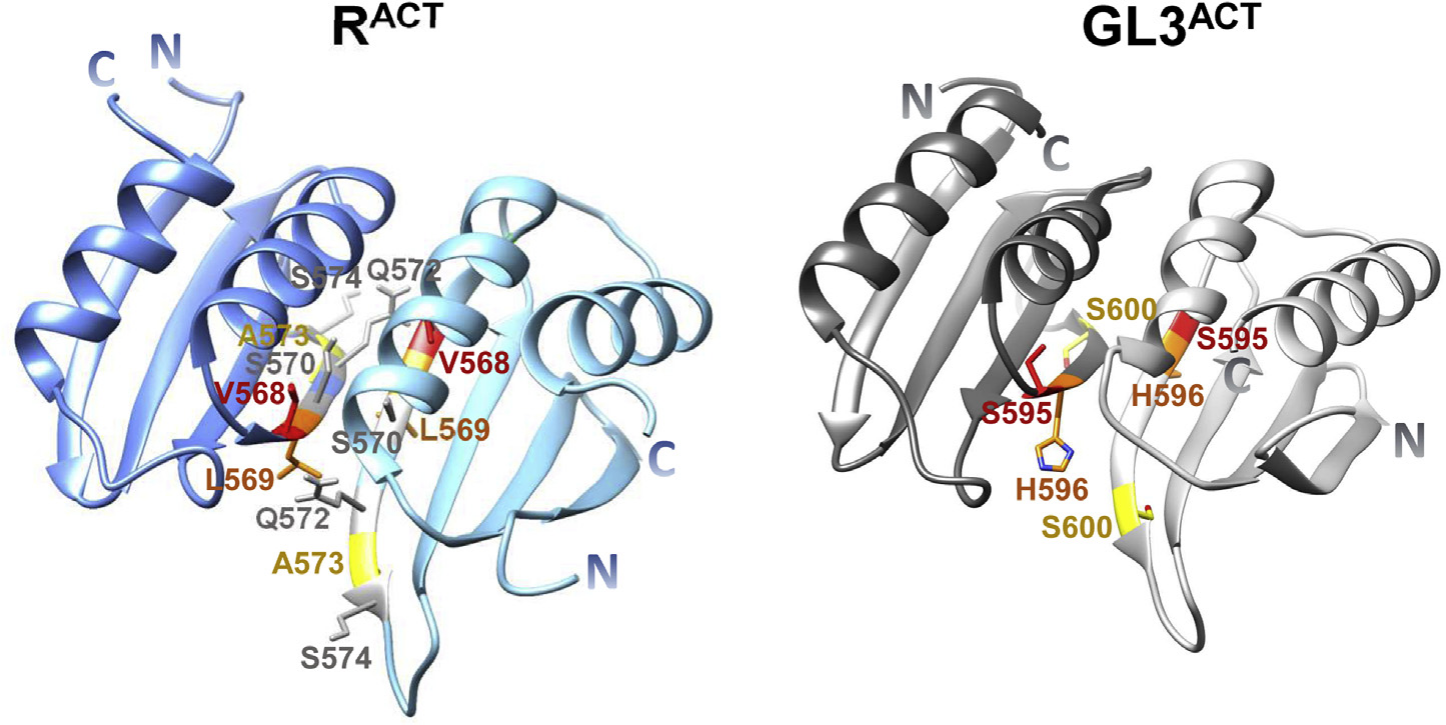
**Predicted structures of R**^**ACT**^**and GL3**^**ACT**^**homodimers in side-by-side configuration.** The structures of R^ACT^ and GL3^ACT^ were predicted based on the structure of the ACT domain of phenylalanine hydroxylase (Protein Data Bank: 5FII). The *red*, *orange*, and *yellow* colors represent the amino acids substituted in our mutational assays. The amino acids (S570, Q572, and S574) of which mutations significantly abolish the ability of the ACT-like domain to dimerize in previous study (26) were indicated in *gray*. ACT, *a*spartate kinase, *c*horismate mutase, and *T*yrA.

To identify the residues that are potentially responsible for the different dimerization strengths of R^ACT^ and GL3^ACT^, we took advantage of homology modeling using phenylalanine hydroxylase (PheH; Protein Data Bank [PDB]: 5FII) as a template, as it showed the highest secondary structure similarity with R^ACT^ and GL3^ACT^. The R^ACT^ and GL3^ACT^ predicted secondary structures showed overall very similar organization, despite having only 33% amino acid identity (Fig. 1*A*). The homology-modeled monomeric R^ACT^ and GL3^ACT^ were then subjected to homodimer predictions using GalaxyHomomer (38) and GalaxyRefineComplex (39). We considered two possible configurations for the respective dimeric forms, side-by-side (Fig. 3) and face-to-face (Fig. S6) arrangements based on what is known on how other ACT domains dimerize (18). Previously, we showed that substitution mutations of S570A, Q572A, and S574A in R^ACT^ abolished its dimerization in Y2H assays (26). The effect of these mutations, which are located on the β2-strand (Figs. 3 and S6), is more consistent with the side-by-side configuration, as these three amino acids would be right at the dimer interface. Based on this potential configuration for the R^ACT^ and GL3^ACT^ dimers, the β2-strand in each is predicted to be important for the interaction. There are three amino acid differences in this region: V568 in R^ACT^ corresponding to S595 in GL3^ACT^, L569 in R^ACT^ corresponding to H596 in GL3^ACT^, and A573 in R^ACT^ corresponding to S600 in GL3^ACT^ (Figs. 1*A* and 3). Significantly, the three amino acids in R^ACT^ are hydrophobic, whereas the corresponding ones in GL3^ACT^ are polar.

**Figure 4:**
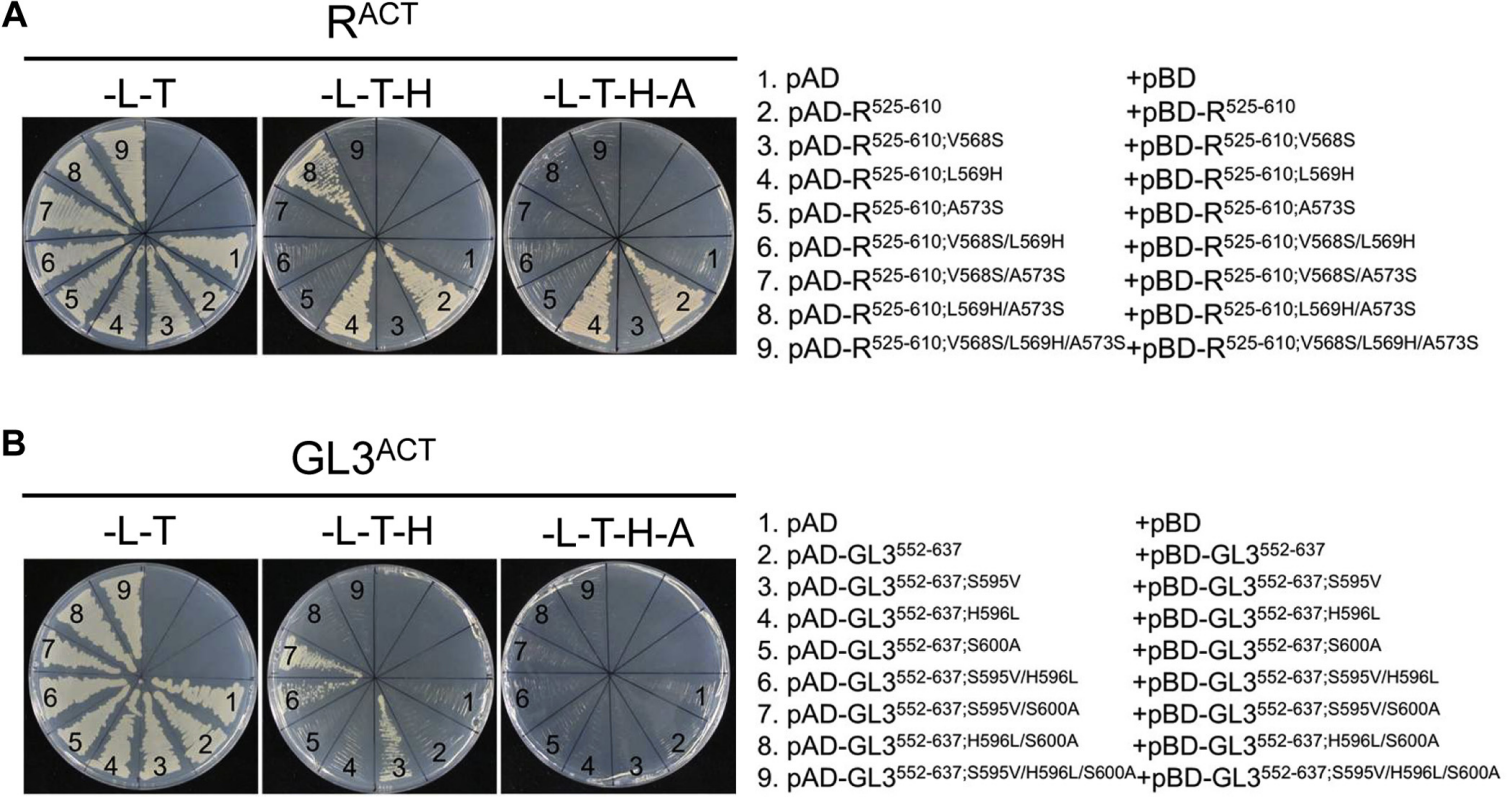
**Effect of mutations on the ability of R**^**ACT**^**and GL3**^**ACT**^**to homodimerize in yeast two-hybrid assays.***A*, homodimerization of R^ACT^ mutants and GL3^ACT^ mutants in yeast. *B*, homodimerization of GL3^ACT^ and GL3^ACT^ mutants in yeast. The R^ACT^ and GL3^ACT^ harboring single, double, or triple substitutions were fused to either the GAL4 DNA-activation domain (pAD) or GAL4 DNA-binding domain (pBD). The dimeric interactions of the R^ACT^ and GL3^ACT^ mutants were examined on the SD media (–L–T, –L–T–H, –L–T–H–A) for 5 days. Autoactivation controls are provided in Figure S1. The empty vector combinations (pAD and pBD represented as #1 in *A* and *B*) were used as negative control. Three independent transformants were analyzed for each plasmid combination corresponding to three biological replicates. SD, synthetically defined.

To determine the contribution of each of these three residues to the different dimerization strengths of R^ACT^ and GL3^ACT^, we made the corresponding single, double, and triple amino acid substitutions in each of the two ACT domains and tested their ability to interact in Y2H assays. When we substituted V568 by S in R^525–610^ (R^525–610;V568S^), dimerization was completely abolished, reflected in no growth in –L–T–H or –L–T–H–A and very low β-galactosidase activity, compared with the robust dimerization of R^525–610^ (Fig. 4*A*, compare #2 and #3; and Fig. S2*A*). A similar dimerization abatement was observed for the A573S substitution (Fig. 4*A*, compare #2 and #5; and Fig. S2*A*). In contrast, the L569H mutation had no effect on the dimerization activity of R^525–610^, as evidenced by the similar growth and β-galactosidase activity with yeast strain harboring the wildtype versions (Fig. 4*A*, compare #2 and #4; and Fig. S2*A*). The double substitutions followed the expected trend, with the V568S/L569H and V568S/A573S showing no R^525–610^ dimerization (Fig. 4*A*, #6 and #7; and Fig. S2*A*). Unexpected was the observation that the yeast strain harboring the L569H/A573S double mutant displayed significantly better interaction than the strain with the single A573S mutant (Fig. 4*A*, compare #5 and #8; and Fig. S2*A*).

The reciprocal residue changes were incorporated into GL3^552–637^ and tested for dimerization using the Y2H assay. Compared with GL3^552–637^, which showed no interaction, yeast cells harboring pAD–GL3^552–637;S595V^ and pAD–GL3^552–637;S595V^ showed growth in –L–T–H, but not in –L–T–H–A (compare #2 and #3 in Fig. 4*B*), consistent with increased β-galactosidase activity (Fig. S2*B*), indicating that the S595V substitution is sufficient to confer the ability of GL3 to dimerize to levels that can now be detected in Y2H assays. No significant interaction was observed for any of the other single or multiple mutations, with the exception of S595V/H596L and S595V/S600A, which showed growth in –L–T–H and moderate β-galactosidase activity (Figs. 4*B* and S2*B*). While the proposed structural models can accurately predict the effect of the other mutations, it fails to explain the ability of the S595V/H596L and S595V/S600A mutants in GL3 to dimerize.

**Figure 5:**
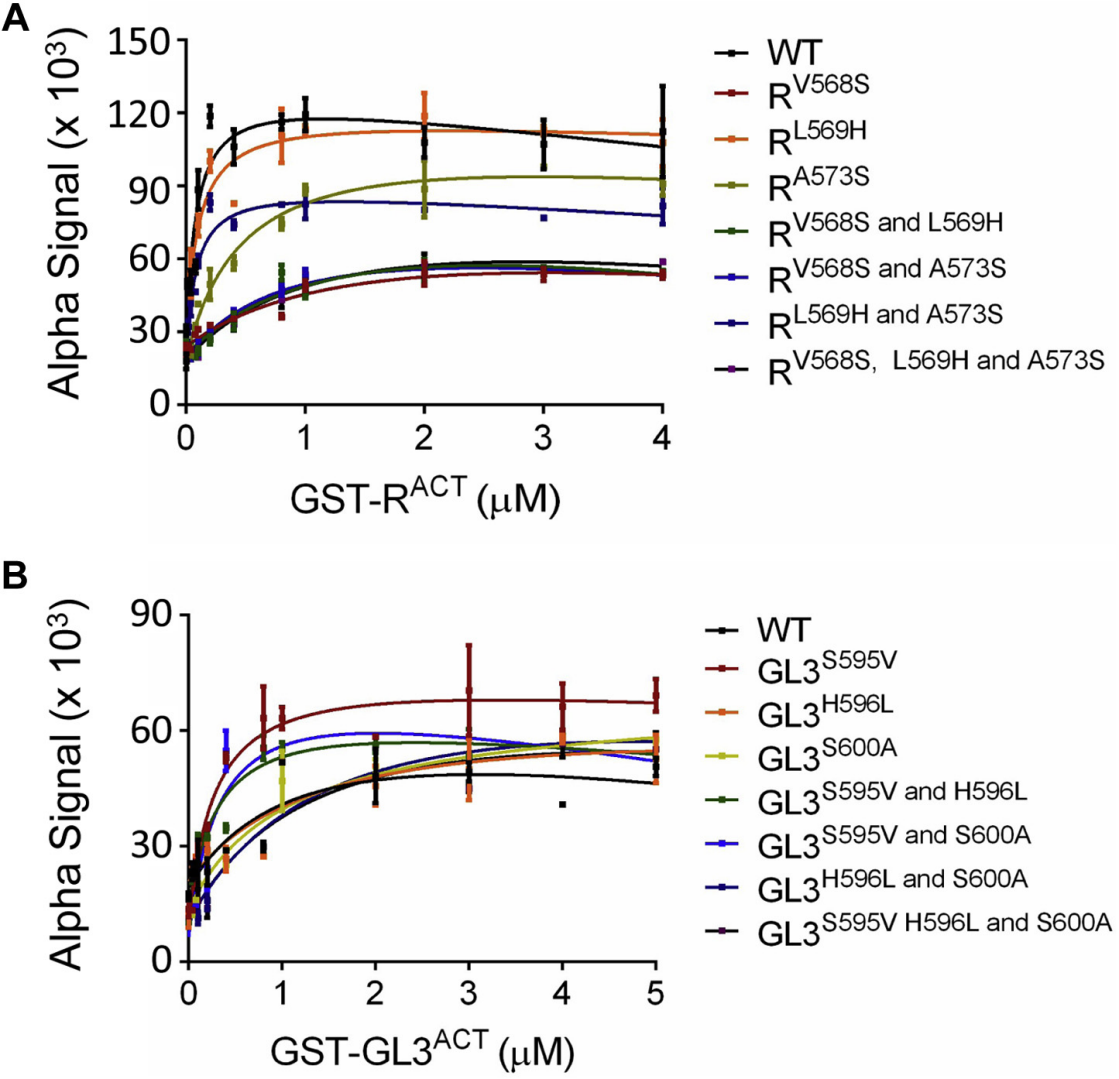
**Equilibrium dissociation constant (*K***_***d***_**) evaluation for R**^**ACT**^**, GL3**^**ACT**^**, and respective mutants.***A*, saturation binding assays with R^ACT^ mutants. *B*, saturation binding assay with GL3^ACT^ mutants. The 100 nM N_6_His-R^ACT^ and 500 nM N_6_His-GL3^ACT^ were incubated with different concentrations of GST-R^ACT^ (0–4 μM) and GST-GL3^ACT^ (0–5 μM) proteins at room temperature for 2 h, before collecting amplified luminescent proximity homogeneous assay responses. The saturation binding curves of R^ACT^ and GL3^ACT^ were obtained by one-site fit model. We show the mean of three biological repeats with two technical repeats each with error bars denoting the standard deviation of the mean. All the data used for the saturation binding curves are provided in Figure S5, and the mean *K*_*d*_ values are shown in Table 1.

To quantitatively evaluate and compare the interaction strengths of the various R and GL3 ACT mutants, *K*_*d*_ values were determined by saturation binding assays using ALPHA and the respective proteins tagged with GST or N_6_His (Fig. 2*A*). The apparent *K*_*d*_ values determined by saturation binding for R^525–610^ were very similar to those determined by competition binding and corresponded to 100 ± 27 nM (competition binding; Fig. 2*B*) and 100 ± 10 nM (saturation binding; Table 1, Figs. 5*A* and S7). Also consistent between both methods, the *K*_*d*_ values for GL3^552–637^ were 1.28 ± 0.17 μM estimated by the competition assay (Fig. 2*C*) and 1.54 ± 0.12 μM by the saturation assay (Table 1, Figs. 5*B* and S7). We then evaluated apparent *K*_*d*_ values for all the mutants analyzed by Y2H. ACT-like domain variants that showed no interaction by Y2H had *K*_*d*_ values that were 400 nM or higher, whereas those that interacted in yeast displayed *K*_*d*_ values in the range of 100 to 400 nM (Fig. 4 and Table 1). Our results indicate that the homodimerization of R^ACT^ and GL3^ACT^ most likely is a consequence of a side-by-side association as found in other ACT domains with a βαββαβ structure (40, 41, 42), in which are the α1-helix and the β2-strand at the interface of the two subunits (Fig. 3).

### Heterodimer formation of subunit interface mutants

The analyses so far involved the formation of homodimers, in which both subunits were identical (*i.e.*, either wild type or mutant). To determine the role of the amino acid residues in the subunit interface identified as important for dimerization, we investigated their role in the formation of heterodimers, in which the subunits harbored different mutations. For this, we combined the various mutants for R^ACT^ and GL3^ACT^ in Y2H assays.

**Figure 6:**
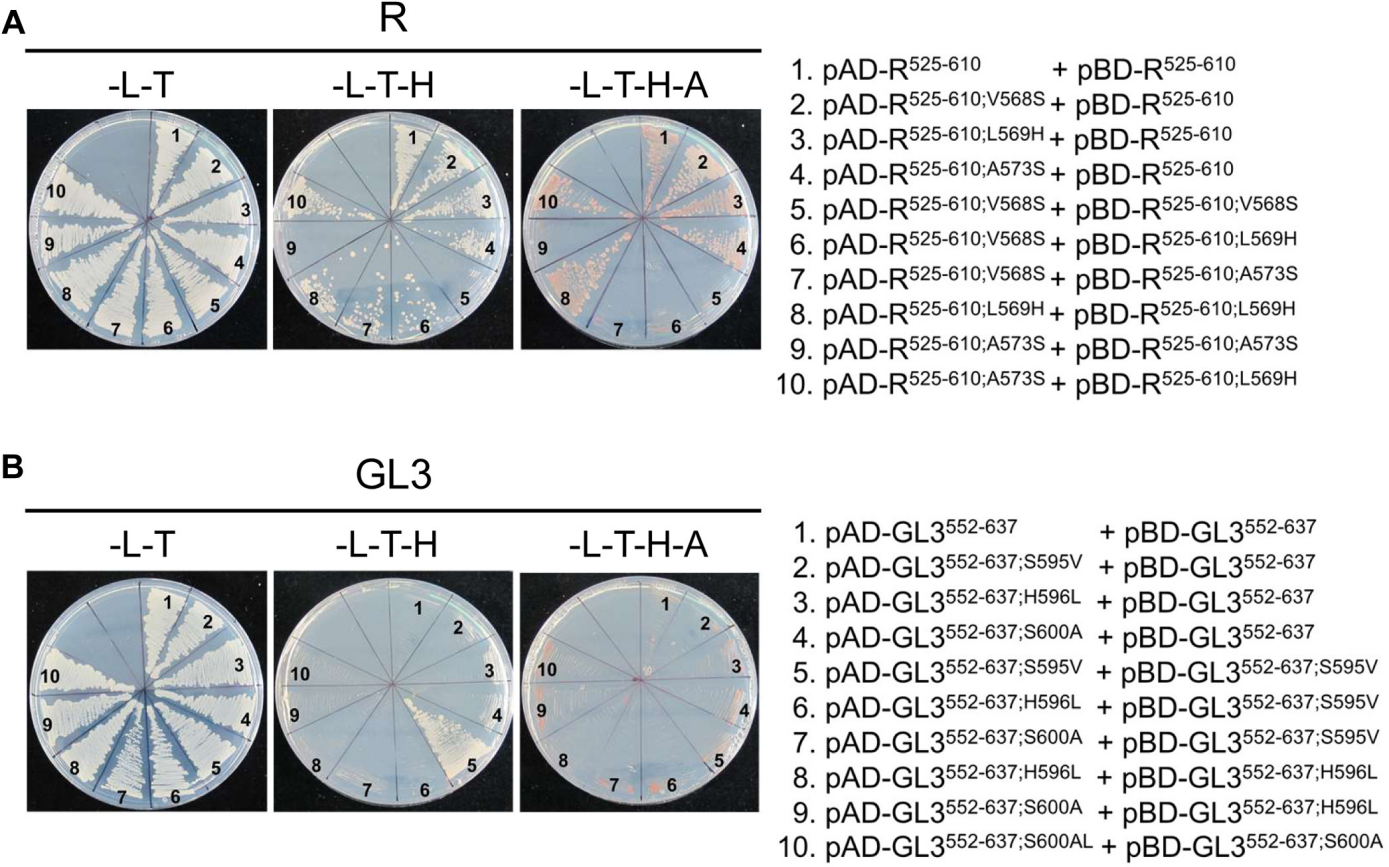
**Effect of mutations on the ability of R**^**ACT**^**and GL3**^**ACT**^**to form heterodimers in yeast two-hybrid assays.***A*, heterodimerization test of R^ACT^ and its mutants in yeast. *B*, heterodimerization test of GL3^ACT^ and its mutants in yeast. The effect of single and multiple amino acid mutations of R^ACT^ and GL3^ACT^ on heterodimer formation was evaluated on selective SD media (–L–T, –L–T–H, –L–T–H–A) for 5 days. SD, synthetically defined.

As we showed before (#3 in Figs. 4*A* and S2*A*), V568S abolishes R^ACT^ dimerization, yet pAD–R^525–610;V568S^ robustly interacts with pBD–R^525–610^ (Fig. 6*A*, compare #2 and #5). A similar situation was observed for R^525–610;A573S^; it is unable to form homodimers (Fig. 4*A*, #5; and Fig. S2*A*), yet it forms robust heterodimers with pBD–R^525–610^ (Fig. 6*A*, compare #4 and #9). When the V568S and A573S mutations are combined in each subunit, heterodimerization continues to happen, yet is somewhat impaired (reflected in heterodimers growing in –L–T–H but not in –L–T–H–A, Fig. 6*A*, #7). The V568S/L569H double mutant of R^ACT^ also formed a heterodimer (Fig. 6*A*, #6), and the *K*_*d*_ value (0.33 ± 0.02 μM) was comparable to that of V568S/A573S (0.27 ± 0.02 μM) in saturation binding assays (Fig. S8). A rather different situation was observed with GL3^ACT^, as none of the heterodimers tested, including those that contained one of the subunits harboring the S595V mutation that allowed dimerization (Fig. 4*B*, #3; and Fig. S2*B*), showed growth (Fig. 6*B*). Taken together, these results highlight the importance of V568 in R^525–610^ and S595 in GL3^552–637^ in establishing the strength of the dimerization. The heterodimer results are consistent with an antiparallel orientation of the dimers, as shown in Figure 3.

### Analysis of other plant ACT domains

Given our results showing the importance of V568 for the dimerization of R^ACT^, we asked how often this residue if present in ACT-like domains associated with bHLH TFs, and whether the presence of this (or similar residue) was correlated with robust homodimer formation.

**Figure 7:**
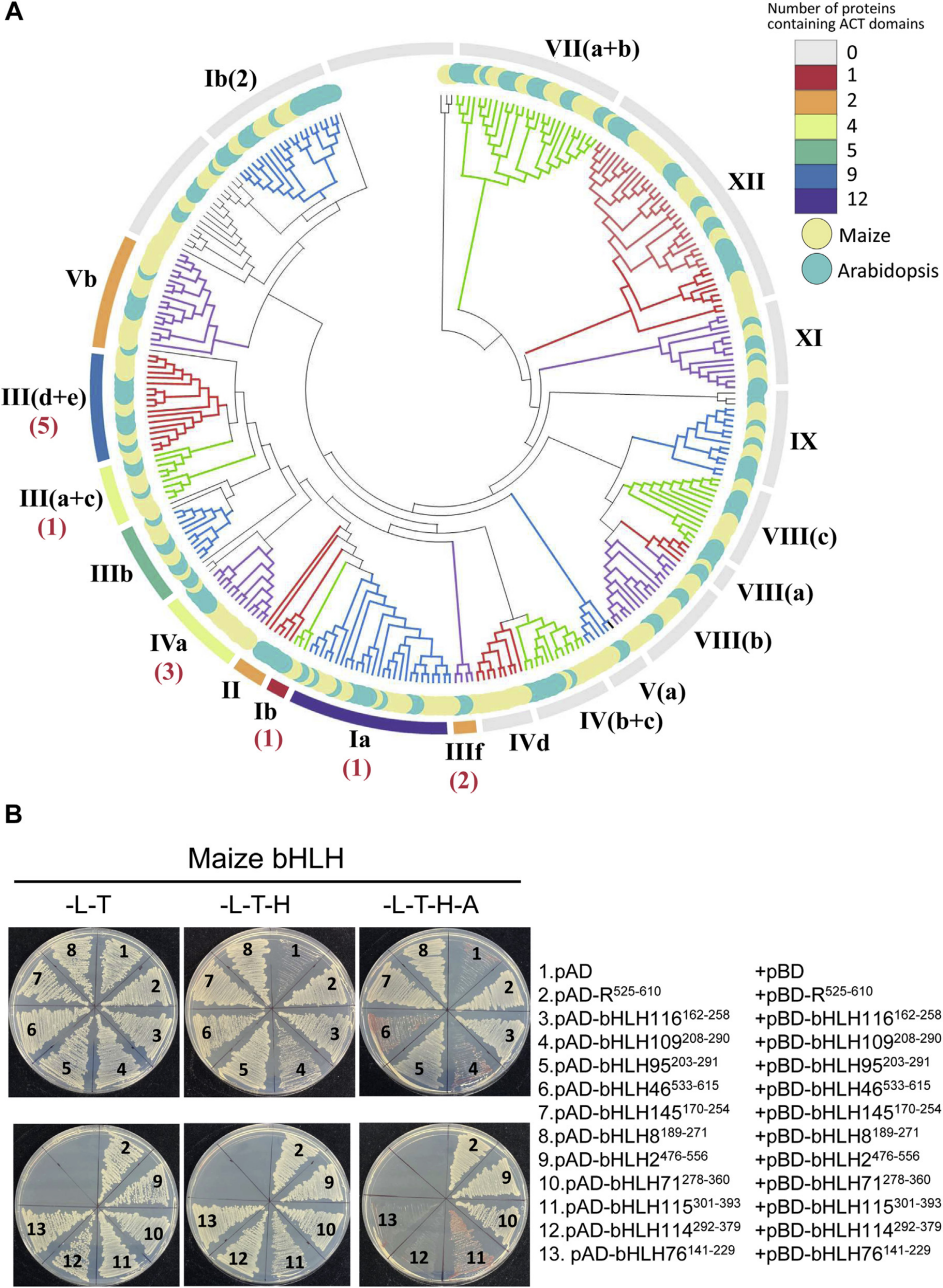
**Analyses of other maize ACT-like domains.***A*, phylogenetic relationship of 175 maize and 137 *Arabidopsis* bHLH TFs. The phylogenetic tree was constructed using the entire amino acid sequence of each of the proteins using maximum likelihood methods with 1000 bootstraps with MEGA7 (59). The maize bHLH subfamilies were assigned according to what was done in *Arabidopsis* TFs of the same clade (7). The subfamilies are indicated by the *lines* in different colors with the respective numbers. The bootstrap support values and taxon names were omitted in the interest of making the illustration legible. The number of ACT-like domains in each subfamily is indicated by the colors in the *outer circle*, and the numbers in *red* indicate how many members of each subfamily were found to homodimerize in yeast two-hybrid (Y2H) experiments. *B*, examples of homodimerization analyses of maize ACT-like domains by Y2H assays. A total 42 of 44 maize bHLH TFs were subjected to the Y2H assay. The interactions were determined on selective SD media (–L–T, –L–T–H, –L–T–H–A). The #1 and #2 indicate the homodimer of R^ACT^ as a positive control and empty vector combination as a negative control, respectively. Autoactivation tests are presented in Figure S9. All the relevant information for the 42 constructs and all Y2H results are presented in Table S1. ACT, *a*spartate kinase, *c*horismate mutase, and *T*yrA; bHLH, basic helix–loop–helix; SD, synthetically defined; TFs, transcription factors.

For this, we first retrieved the sequence of all 175 maize bHLH factors from GRASSIUS (grassisus.org) (43). Because of the low amino acid sequence conservation between ACT-like domains (13, 14), we determined whether they harbored an ACT domain by predicting secondary structures using PSIPRED (44). We found that 44 of 175 bHLH factors had an ACT-like domain, a frequency similar to what was previously determined for *Arabidopsis* (13), and this domain is always located at the C-terminus, as is the case for R and GL3 (Fig. 1*A*). ACT-like domains characterize members of bHLH subfamilies I, II, III, IV, and Vb (Table S1 and Fig. 7*A*), as was previously determined for *Arabidopsis* (13). These results demonstrate that the origin and distribution of ACT-like domains in bHLH TFs precedes the divergence of monocot and dicot.

To determine how many of these maize ACT-like domains formed homodimers detectable by Y2H assays, we cloned 42 of 44 in the pAD and pBD Y2H vectors and analyzed their ability to form homodimers. Of the 42 ACT-like domains tested, 12 (including R) were capable of homodimerizing (Figs. 7*B* and S9). All the ACT-like domains displaying robust dimerization in the Y2H assays belonged to bHLH subfamilies III(d+e), IIIf (to which R and GL3 belong), and IVa (Fig. 7*A*, dimerization indicated by numbers in *red*). The remaining 30 ACT-like domains that showed no yeast growth in selective media are either incapable of dimerizing, or the dimerization is too weak (*K*_*d*_ >400 nM) to be determined by Y2H assays, as we established for GL3^ACT^.

We then investigated whether the presence of Val (or similar aliphatic amino acid) at the equivalent position of V568 in R^ACT^ was associated with the strength of the dimerization. The alignment of the 42 maize ACT-like domains showed that this position is occupied by Val, Ile, Leu, or Ala (and an exceptional Pro). However, the presence of a Val was not indicative of whether the respective ACT-like domain would dimerize in Y2H or not (Fig. S10). Taken together, our results demonstrate that while V568 is clearly essential for robust dimerization in R, the presence of this residue is not an indication of whether an ACT-like domain will dimerize in Y2H assays or not. Moreover, our results suggest that strong dimerization is a property of only a subset of maize ACT-like domains associated with bHLH TFs.

## Discussion

We used homology modeling to predict the structure of the ACT-like domains of R and GL3. We show that both ACT-like domains can dimerize, albeit with very different affinities, evidenced by over an order of magnitude difference in their *K*_*d*_ values. This knowledge was then applied to identify key subunit interface residues responsible for the different interaction strengths. The R^ACT^ β-strand that provides the subunit interface is characterized by more aliphatic residues, whereas the GL3^ACT^ corresponding region contains polar residues at the same locations. Indeed, the V568S and A573S substitutions completely abolish R^ACT^ dimerization in Y2H assays (Figs. 4 and S2) reflected in a significant reduction in the affinity of the interaction (Figs. 4 and S2, Table 1), underscoring the importance of this β2-strand in subunit interaction. Strikingly, the S595V substitution in GL3^ACT^ (the corresponding substitution to V568S in R^ACT^), but not the S600A (corresponding to A573S in R^ACT^), confers GL3^ACT^ the ability to dimerize with an affinity comparable of that of R^ACT^ (Figs. 4 and S2, Table 1). The position of V568 in our models coincides with the position of I65 in the structure of the ACT domain of PheH (PDB: 2PHM), residues that were determined to be important in maintaining the hydrophobic packing of the protein (Fig. S11) (45).

These results are significant from several perspectives. Dimerization of the ACT-like domain in R was proposed to inhibit dimerization and DNA binding of the adjacent bHLH motif (26). Thus, the presence of strong ACT dimerization could be used as an indication of whether the DNA-binding activity of the bHLH is regulated by the ACT-like domain or not. GL3 is an *Arabidopsis* R ortholog and also controls anthocyanin accumulation (32). However, GL3 is one of three *Arabidopsis* bHLH factors that can control anthocyanin accumulation, the other two being TT8 and EGL3 (32). Both GL3 and EGL3 have Ser at the V568 position, whereas TT8 has Thr. This contrasts dramatically with all the maize ACTs that have an aliphatic residue in this position (Fig. S10). To determine if this is a particularity of the *Arabidopsis* anthocyanin regulators, we retrieved the sequence of the ACT domain from the predicted anthocyanin regulators from many plant species (Fig. S12). Interestingly, the identity of the residue at the V568 position can clearly distinguish between different subclades, including a MYC1/R subclade with mainly Val/Ala at this position, a GL3 subclade with Ser/Cys at this position, and the TT8 group with occasionally Val/Ile but largely a Thr for Brassicaceae family (Fig. S12). To what extent these variations reflect differences in the regulatory mechanisms in which these TFs participate remains to be investigated.

The possibility to modulate the dimerization strength of ACT-like domains, for example, by replacing the residue at the equivalent V568 position with Ser/Cys, opens interesting opportunities to alter the regulatory activity of bHLH factors harboring these structurally conserved domains. It remains to be determined if the dimerization of ACT-like domains is affected by the interaction with small molecules, as is the case for many ACT domains (18). But if these were the case, it is possible to envision a situation in which the regulation of specific metabolic or developmental pathways is made more or less responsive to particular ligands, with potential in agriculture.

There are many different methods to assay PPIs, each with its strengths and limitations (46, 47, 48). By and large, it is not known what is the limit in the interaction strength that permits to detect PPIs by the Y2H method. Studies conducted on the interaction of retinoblastoma to other proteins suggested that the weakest binding constant that would give a positive by the Y2H assay is around 1 μM (48). While this certainly depends on many factors, including the specific proteins tested and their level of expression in yeast, our results are largely in agreement and indicate that interactions involving the R and GL3 ACT domains with *K*_*d*_ values above 400 nM will be difficult to assess using Y2H. Moreover, the difference in *K*_*d*_ value ranges between hybrids that support growth in –L–T–H (∼110–340 nM) and those that support growth in –L–T–H–A (<160 nM) is not very different. These results indicate that, while for extreme cases, the additional selection furnished by omitting adenine from the media or adding 3-AT can help discriminate strong *versus* weak interactions (49, 50), there is a range of interaction strengths for which the distinction is less clear.

The overall results provide novel functional insights into how structurally conserved, yet sequence divergent, ACT-like domains present in one of the largest families of plant TFs dimerize. Our studies bring forward a model for the dimerization of these ACT-like domains, models that are consistent with the mutational data. The possibility to alter the strength of the interactions by switching single conserved residues provides a powerful tool to elucidate the function of these domains *in vivo*.

## Experimental procedures

### Plasmids

The gateway entry clones harboring ACT-like domains were synthesized by GeneArt Gene Synthesis (ThermoFisher Scientific) or generated by PCR from full-length open reading frame clones generated by the Maize TFome project (51). The pENT constructs were recombined into the pDEST17 and pDEST15 (ThermoFisher Scientific) for recombinant protein expression and purification. The pAD–GAL4–GWC1 and pBD–GAL4–GWC1 vectors (52), referred to as pAD and pBD, were used for Y2H assays. For untagged ACT-like domains of R and GL3, the corresponding regions were inserted into the BamHI/HindIII sites of pET28–SUMO (53) using the following primer sets. R: forward 5′-CACCGGATCC GACGCCGGCACCAGCAACGTCA-3′, reverse 5′-AAGCTTTCACCGCTTCCCTATAGCTTTGCGA-3′; GL3: forward 5′-ATGGATCCTTTACTGGTTTAACCGATAA-3′ reverse 5′-GCAAGCTT TCAACAGATCCATGCAACCC-3′.

### Y2H assays

We used the PJ69.4a yeast strain that harbors integrated in the genome the *HIS3* and *ADE2* selectable genes controlled by GAL4-binding sites (37). The pAD and pBD constructs were transformed into PJ69.4a by the lithium chloride method with a slight modification (54). Briefly, yeast cells (absorbance at 600 nm ≈ 0.4) were resuspended in 100 mM lithium acetate, 50% PEG, and 30 μg/ml salmon sperm DNA (Invitrogen), and incubated with 500 ng of pAD and pBD plasmid at 30 °C for 30 min followed by 42 °C for 20 min and incubated 30 °C for 1 h. To select yeast colonies, the transformed yeast cells were cultured on an SD medium lacking Leu and Trp (–L–T) at 30 °C for 3 days. The positive colonies were subcultured on SD–Leu/–Trp (SD–L–T), SD–His/–Leu/–Trp (SD–H–L–T), and SD–Ade/His/–Leu/–Trp (SD–A–H–L–T) to test physical interactions. The strength of homodimerizations in the yeast cells was quantitatively measured using the β-galactosidase assay (55) The crude extracts of the yeast cells were incubated with 0.8 mg/ml *o*-nitrophenyl-*β*-d-galactopyranoside (Sigma), and the resultant absorbance values at 420 nm were normalized to protein concentration, evaluated by Bradford assays (56). Arbitrary β-galactosidase units were calculated as follows: (absorbance at 420 nm × 1.7)/(0.0045 × time of incubation × volume of extract × protein concentration in mg/ml). Yeast growth and β-galactosidase assays were conducted in three biological replicates, each obtained from independent transformants.

### Recombinant protein purification

The ACT-like domains of R and GL3 were fused to the C terminus of His_6_ or GST and transformed into the BL21(DE3) strain. The cells were cultured in 100 ml (absorbance at 600 nm ≈ 0.4) at 37 °C, and induced by 0.6 mM IPTG (final concentration). After incubation at 37 °C for 2 h, the cells were harvested and resuspended in 5 ml of modified PBS buffer (500 mM NaCl,10 mM Na_2_HPO_4_, 2 mM KH_2_PO_4_, and 0.05% Triton X-100) followed by sonication (Misonix Ultrasonic Liquid Processors S-4000). The cell lysate was centrifuged at 3500*g* for 20 min and filtered through Miracloth (Calbiochem).

For poly-His-tagged protein purification, the cell filtrate was incubated with 200 μl of a 50% (w/v) slurry of nickel–nitrilotriacetic acid agarose (ThermoFisher Scientific), equilibrated with PBS buffer at 4 °C for 1 h, and washed with 5 ml of PBS buffer containing 50 mM imidazole. The resin was eluted by three subsequent washes of 500 μl each of 400 mM imidazole in PBS buffer. For GST-tagged protein purification, 200 μl of glutathione–agarose beads (Roche) in PBS buffer were mixed with the cell filtrate and incubated at 4 °C for 1 h. The resin-bound protein was rinsed with 5 ml PBS buffer and eluted three times with 500 μl each of PBS buffer containing 10 mM reduced glutathione (pH 8.0). To purify the untagged ACT-like domains of R and GL3, the pET28–SUMO constructs were transformed into BL21(DE3). The bacterial cells were cultured in 500 ml until an absorbance at 600 nm ≈ 0.4 and further incubated in 0.6 mM IPTG at 16 °C for 12 h. The harvested cells were resuspended in buffer containing 10 mM Tris–HCl, 150 mM NaCl, 0.5 mM Tris(2-carboxyethyl)phosphine (TCEP)–HCl, and 5 mM imidazole followed by sonication and centrifugation. Cleared supernatants were bound to the columns by gravity flow and washed two times with 50 ml buffer (50 mM Tris–HCl and 350 mM NaCl plus 10, 30, or 50 mM of imidazole, pH 8.0). The elution was carried out four times with 10 ml buffer (50 mM Tris–HCl, 350 mM NaCl, and 250 mM imidazole, pH 8.0). The SUMO tag was removed from the eluted protein during the dialysis in the buffer supplemented with 350 mM NaCl, 50 mM Tris–HCl, 0.5 mM TCEP–HCl (pH 8.0), and in-house produced N_6_His-SUMO protease (protease:protein ratio = 1:16) for 2 h at room temperature. The suspension was then transferred to the second nickel–nitrilotriacetic acid column, and the cleaved protein was eluted with 2× column volume of buffer containing 350 mM NaCl, 50 mM Tris–HCl, 0.5 mM TCEP–HCl, and 10 mM imidazole (pH 8.0). All purified proteins were analyzed on SDS-PAGE (15%, 37.5:1 acrylamide:bisacrylamide; BioRad) after Coomassie brilliant blue (G-250; Thermo Scientific) staining.

### Western blot analysis

Total proteins were extracted from the yeast cells by the urea/SDS method according to the manufacturer's protocol (Clontech) without modifications. The extracted proteins were quantified by the Bradford assay (Bio-Rad), and 20 μg of each extract were loaded onto 15% SDS-PAGE gel (37.5:1 acrylamide/bisacrylamide) followed by transfer to a polyvinylidene fluoride membrane at 100 V for 75 min. Blocking was done with 5% fat-free milk in 1× Tris-buffered saline with Tween-20 (10 mM Tris–Cl [pH 8.0], 150 mM NaCl, and 0.01% Tween-20) at 4 °C overnight. Membranes were probed with GAL4 AD monoclonal antibody in a dilution of 1:2500 (630402; Clontech) at room temperature for 1 h and rinsed three times with 5% fat-free milk in 1× Tris-buffered saline with Tween-20 at room temperature, each for 10 min. For the secondary antibody, we used a 1:10,000 dilution of a antimouse antibody coupled to IRDye 800CW (LI-COR Biosciences), which was added to the blot and washed for 1 h after incubation, as described for the primary antibody. Western blots were visualized using a Sapphire Biomolecular imager (Azure Biosystems).

### ALPHA

Dimerization kinetics were determined by measuring *K*_*d*_ values using the ALPHA assay on a Synergy Neo2 Hybrid Multi-Mode Reader (BioTek) according to the manufacturer's PerkinElmer protocol. For the competition binding assay, 100 nM of N_6_His-R^ACT^ and GST-R^ACT^ and 100 nM of N_6_His-GL3^ACT^ and GST-GL3^ACT^ were combined with different concentrations of untagged R^ACT^ (0–8 μM) and GL3^ACT^ (0–10 μM) and incubated for 2 h at room temperature. Saturation binding assays were performed by incubating different concentration of His-tagged R^ACT^ (0–4 μM) and GL3^ACT^ (0–5 μM) proteins with 100 nM GST-tagged R^ACT^ or 500 nM GL3^ACT^. All protein mixtures were further incubated with Nickel Chelate Alpha Donor beads (20 μg/ml; PerkinElmer) and anti-GST AlphaLISA Acceptor beads (20 μg/ml; PerkinElmer) at room temperature for 2 h. A total of 40 μl of final mixtures were transferred into white 384-well OptiPlate (PerkinElmer), and the signal was read in the Alpha-compatible reader (Biotek). Dissociation constants obtained from both assays were calculated by fitting with one site fit model in GraphPad Prism, version 6.0 (GraphPad Software, Inc).

### Protein structure prediction

Protein monomer secondary structures of GL3^552–637^ and R^525–610^ were predicted using I-TASSER (57). The homodimer structure was estimated by GalaxyHomomer (38) and GalaxyRefineComplex (39). The secondary structures of R and GL3 were obtained based on the ACT domain of PheH (PDB: 5FII).

### Identification of bHLH TFs containing ACT-like domains in maize and phylogenetic reconstructions

The sequences for all maize bHLH TFs were retrieved from GRASSIUS (43). The maize bHLH TFs were aligned with *Arabidopsis* bHLH TFs by MUSCLE (58) to determine subgroup. The phylogenetic tree including all bHLH TFs of *Arabidopsis* and maize was constructed by maximum likelihood method with 1000 bootstrap replicates in MEGA7 (59).

The bHLH TFs containing ACT-like domain were selected on the basis of the presence of ββαββα by PSIPRED (44). The amino acid sequences and secondary structures were aligned to each other by T-Coffee (http://tcoffee.crg.cat/). The amino acid variation was determined by WebLogo, version 3 (https://weblogo.berkeley.edu/logo.cgi). The sequences used in this study are provided in Table S1.

GL3- and TT8-like proteins in 15 different species were isolated from Pytozome (version 12.1; https://phytozome.jgi.doe.gov/pz/portal.html) and Phylogenes (http://www.phylogenes.org/). The sequences were aligned with MUSCLE, and the phylogenetic tree was built by the neighbor joining method with 1000 bootstraps in MEGA7 (59). Secondary structures of the proteins were evaluated as described previously.

## Data availability

All data are contained within the article.

## Supporting information

This article contains supporting information (26, 37).

## Conflict of interest

The authors declare that they have no conflicts of interest with the contents of this article.
