# Supplementary material for: A hydrophobic residue stabilizes dimers of regulatory ACT-like domains in plant basic helix–loop–helix transcription factors
Source: J Biol Chem. 2021 Apr 24;296:100708. doi: 10.1016/j.jbc.2021.100708 (PMC8202348; doi:10.1016/j.jbc.2021.100708)
Supplement: Figures S1 to S12 [file mmc1.pdf]

Supporting Information for:

Identification of residues involved in the dimerization strength of ACT domains of plant bHLH transcription factors

Yun Sun Lee[1], Edian Andres Herrera Tequia[1], Jagannath Silwal[1∮] , James H. Geiger[2], and Erich Grotewold[1*]

[1]Department of Biochemistry & Molecular Biology, and [2]Department of Chemistry, Michigan State University, East Lansing, MI 48824

[∮]Current address: Veterinary Medicine Research and Development, Zoetis Inc, Kalamazoo, MI 49007

[*]To whom correspondence should be addressed: Erich Grotewold: Department of Biochemistry & Molecular Biology, Michigan State University, 603 Wilson Rd, Rm. 212 Biochemistry Bldg., East Lansing, MI 48824-6473, USA; grotewol@msu.edu; Tel. (517) 353-1425.

A

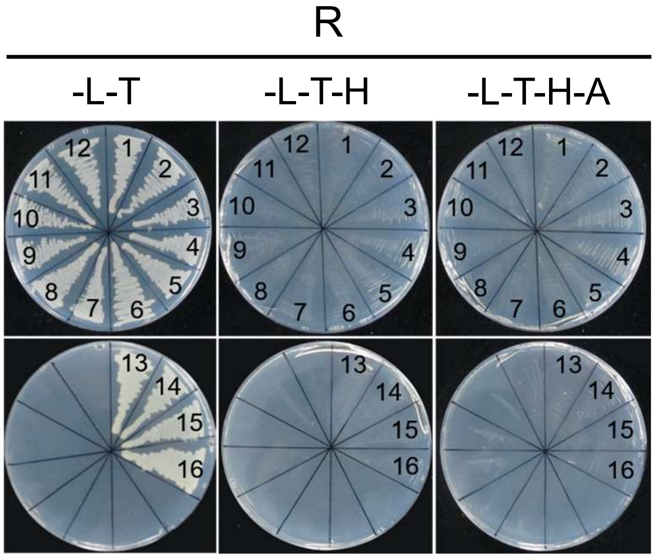

1. pAD-R$^{525\text{-}610}$ + pBD
2. pAD + pBD-R$^{525\text{-}610}$
3. pAD-R$^{525\text{-}610;V568S}$ + pBD
4. pAD + pBD-R$^{525\text{-}610;V568S}$
5. pAD-R$^{525\text{-}610;L569H}$ + pBD
6. pAD + pBD-R$^{525\text{-}610;L569H}$
7. pAD-R$^{525\text{-}610;A573S}$ + pBD
8. pAD + pBD-R$^{525\text{-}610;A573S}$
9. pAD-R$^{525\text{-}610;V568S/L569H}$ + pBD
10. pAD + pBD-R$^{525\text{-}610;V568S/L569H}$
11. pAD-R$^{525\text{-}610;V568S/A573S}$ + pBD
12. pAD + pBD-R$^{525\text{-}610;V568S/A573S}$
13. pAD-R$^{525\text{-}610;L569H/A573S}$ + pBD
14. pAD + pBD-R$^{525\text{-}610;L569H/A573S}$
15. pAD-R$^{525\text{-}610;V568S/L569H/A573S}$ + pBD
16. pAD + pBD-R$^{525\text{-}610;V568S/L569H/A573S}$

B

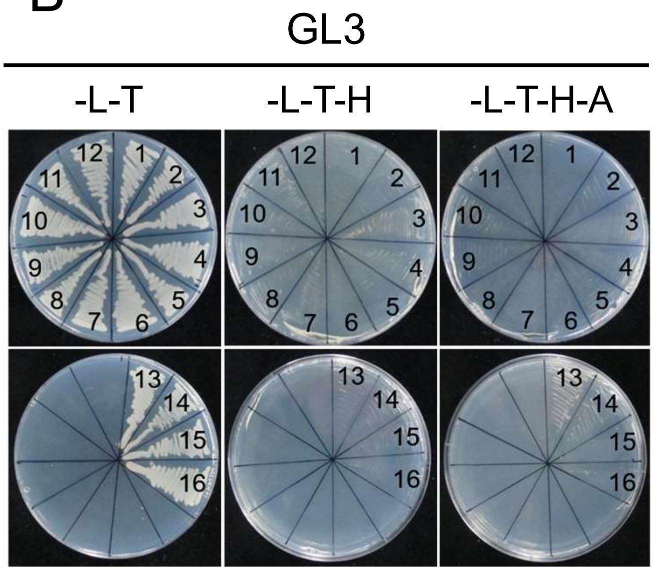

1. pAD-GL3$^{552\text{-}637}$ + pBD
2. pAD + pBD-GL3$^{552\text{-}637}$
3. pAD-GL3$^{552\text{-}637;S595V}$ + pBD
4. pAD + pBD-GL3$^{552\text{-}637;S595V}$
5. pAD-GL3$^{552\text{-}637;H596L}$ + pBD
6. pAD + pBD-GL3$^{552\text{-}637;H596L}$
7. pAD-GL3$^{552\text{-}637;S600A}$ + pBD
8. pAD + pBD-GL3$^{552\text{-}637;S600A}$
9. pAD-GL3$^{552\text{-}637;S595V/H596L}$ + pBD
10. pAD + pBD-GL3$^{552\text{-}637;S595V/H596L}$
11. pAD-GL3$^{552\text{-}637;S595V/S600A}$ + pBD
12. pAD + pBD-GL3$^{552\text{-}637;S595V/S600A}$
13. pAD-GL3$^{552\text{-}637;H596L/S600A}$ + pBD
14. pAD + pBD-GL3$^{552\text{-}637;H596L/S600A}$
15. pAD-GL3$^{552\text{-}637;S595V/H596L/S600A}$ + pBD
16. pAD + pBD-GL3$^{552\text{-}637;S595V/H596L/S600A}$

Figure S1. Autoactivation of wild-type and mutant ACT-like domains of R and GL3 in yeast.

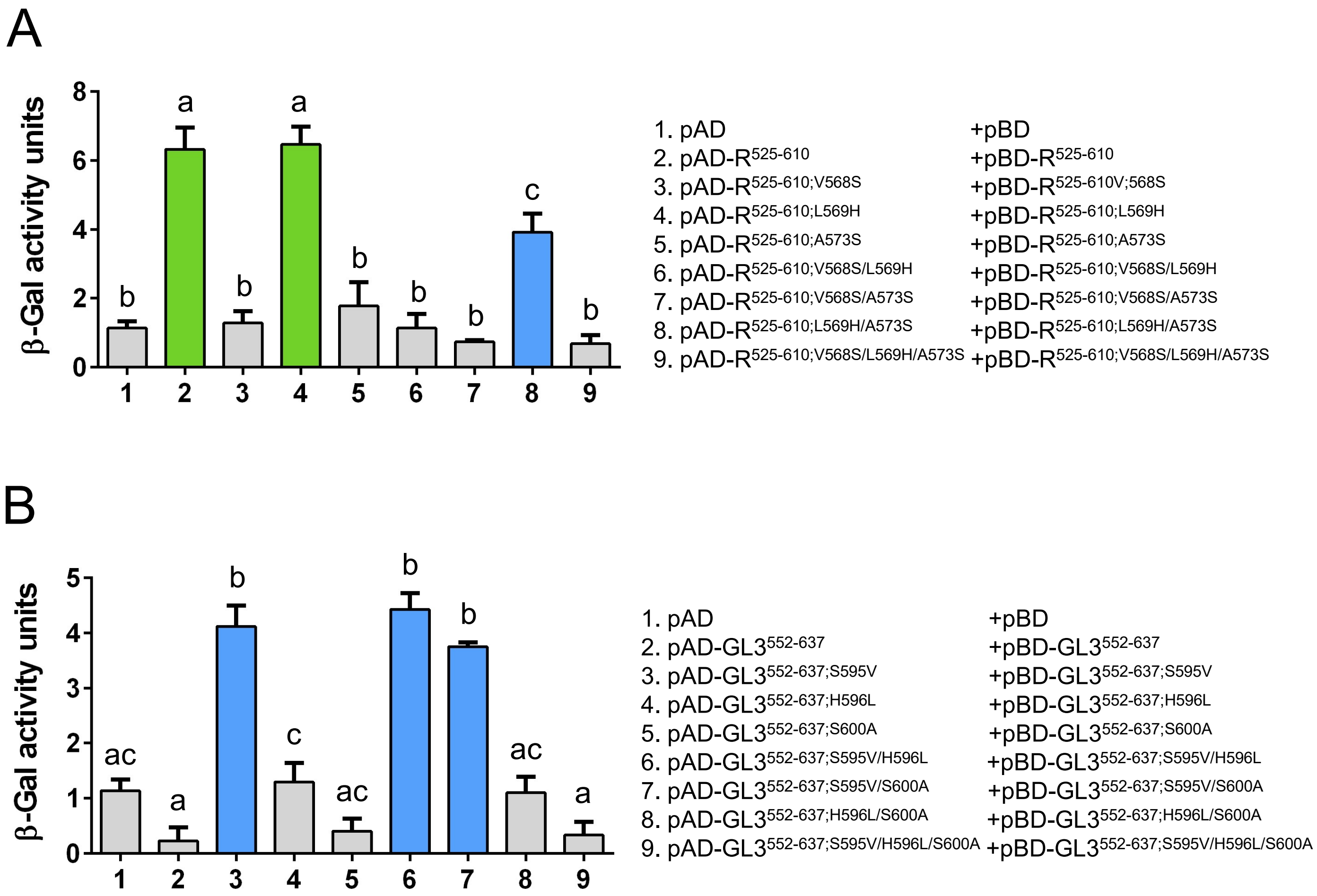

Figure S2. β-Galactosidase activity measurement of the homodimerization of R^{ACT} and GL3^{ACT} in yeast two-hybrid assays.

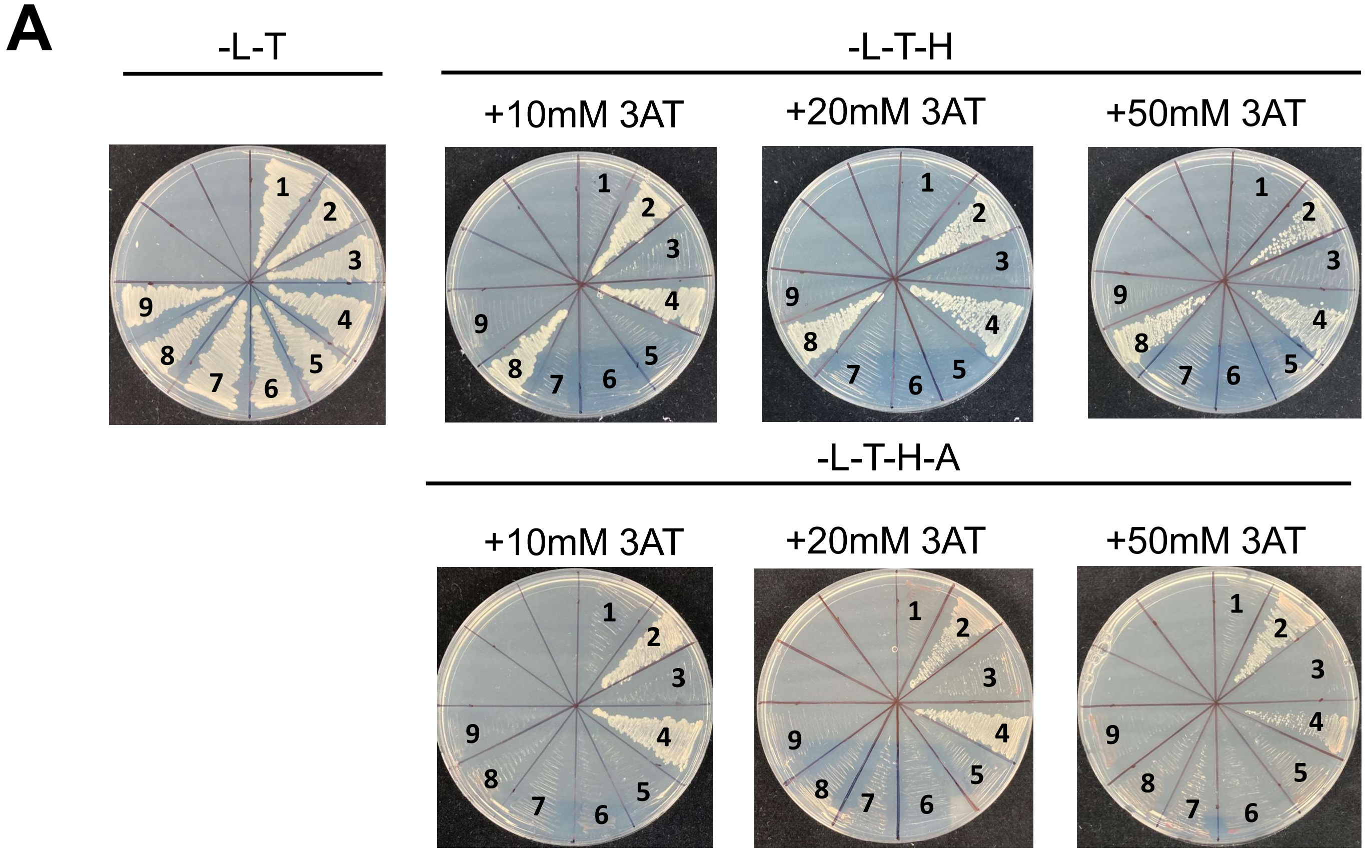

1. pAD +pBD
2. pAD-R$^{525-610}$ +pBD-R$^{525-610}$
3. pAD-R$^{525-610;V568S}$ +pBD-R$^{525-610;V568S}$
4. pAD-R$^{525-610;L569H}$ +pBD-R$^{525-610;L569H}$
5. pAD-R$^{525-610;A573S}$ +pBD-R$^{525-610;A573S}$
6. pAD-R$^{525-610;V568S/L569H}$ +pBD-R$^{525-610;V568S/L569H}$
7. pAD-R$^{525-610;V568S/A573S}$ +pBD-R$^{525-610;V568S/A573S}$
8. pAD-R$^{525-610;L569H/A573S}$ +pBD-R$^{525-610;L569H/A573S}$
9. pAD-R$^{525-610;V568S/L569H/A573S}$ +pBD-R$^{525-610;V568S/L569H/A573S}$

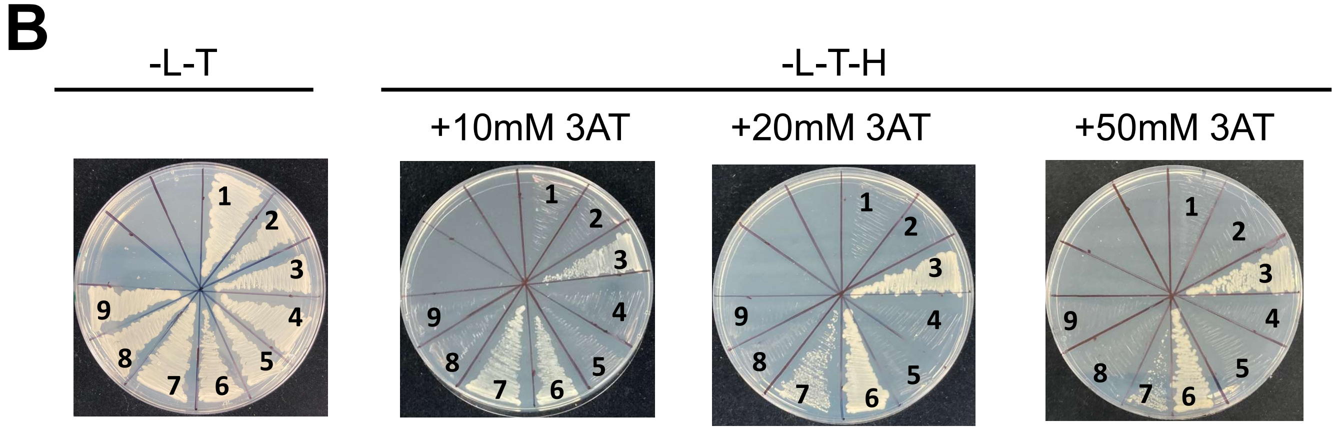

1. pAD +pBD
2. pAD-GL3$^{552-637}$ +pBD-GL3$^{552-637}$
3. pAD-GL3$^{552-637;S595V}$ +pBD-GL3$^{552-637;S595V}$
4. pAD-GL3$^{552-637;H596L}$ +pBD-GL3$^{552-637;H596L}$
5. pAD-GL3$^{552-637;S600A}$ +pBD-GL3$^{552-637;S600A}$
6. pAD-GL3$^{552-637;S595V/H596L}$ +pBD-GL3$^{552-637;S595V/H596L}$
7. pAD-GL3$^{552-637;S595V/S600A}$ +pBD-GL3$^{552-637;S595V/S600A}$
8. pAD-GL3$^{552-637;H596L/S600A}$ +pBD-GL3$^{552-637;H596L/S600A}$
9. pAD-GL3$^{552-637;S595V/H596L/S600A}$ +pBD-GL3$^{552-637;S595V/H596L/S600A}$

Figure S3: Effects of 3-AT on dimerization detected by yeast two-hybrid assays.

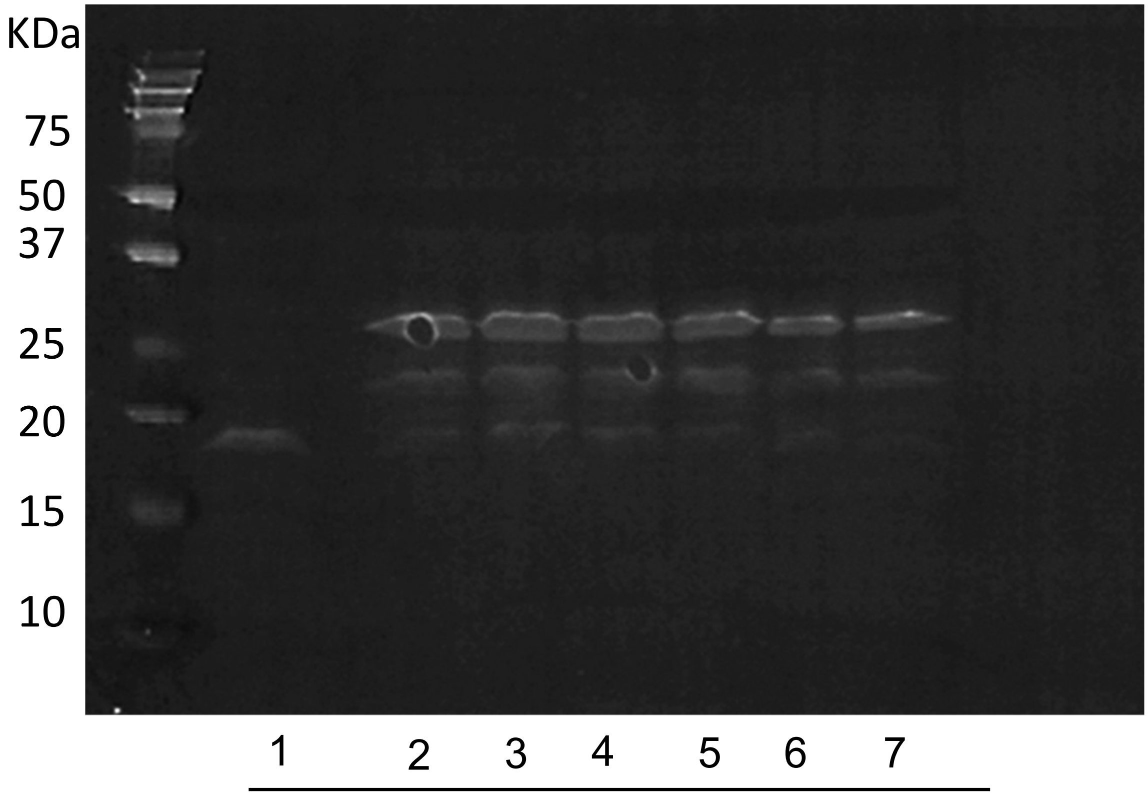

GAL4-AD fused to R and GL3 on 15 % acrylamide gel

Lane 1: AD
2: AD-R$^{525-610}$
3: AD-R$^{525-610;V568S}$
4: AD-GL3$^{552-637}$
5: AD-GL3$^{552-637;S595V}$
6: AD-GL3$^{552-637;H596L}$
7: AD-GL3$^{552-637;S600A}$

Figure S4. Expression of AD-R^{ACT}, AD-GL3^{ACT}, and respective mutants in yeast cells.

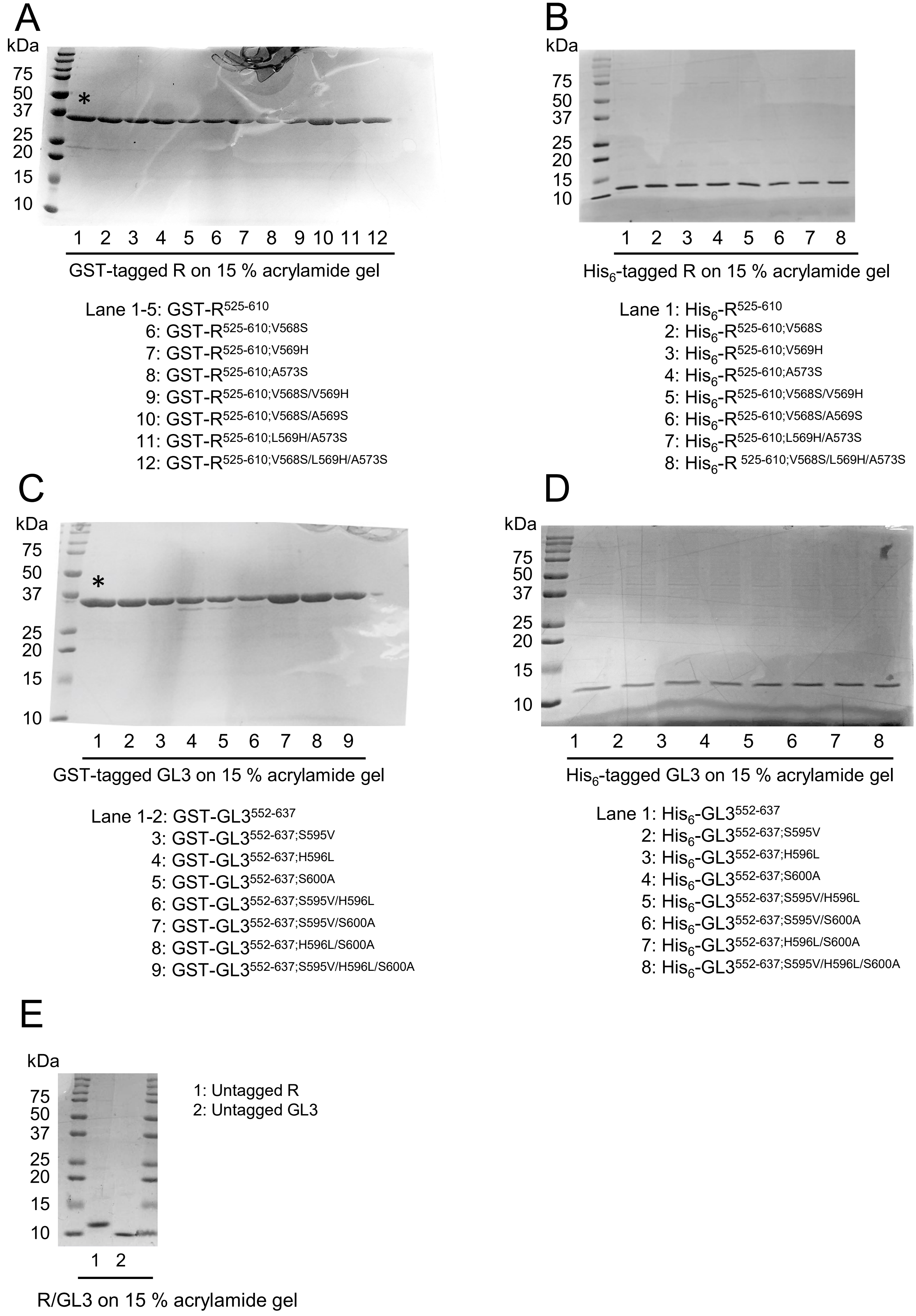

Figure S5. Purified recombinant R^{ACT} and GL3^{ACT} used in Alpha assays.

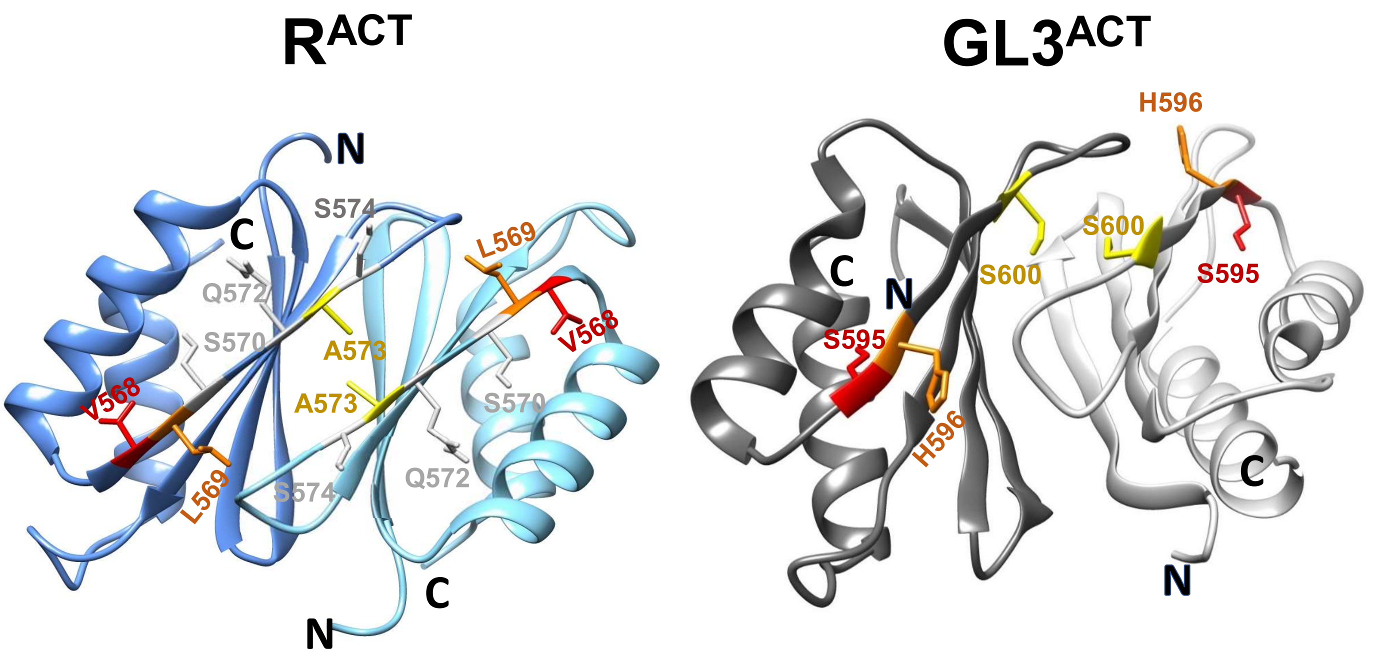

Figure S6. Predicted structures of the R^{ACT} and GL3^{ACT} homodimers in a face-to-face arrangement.

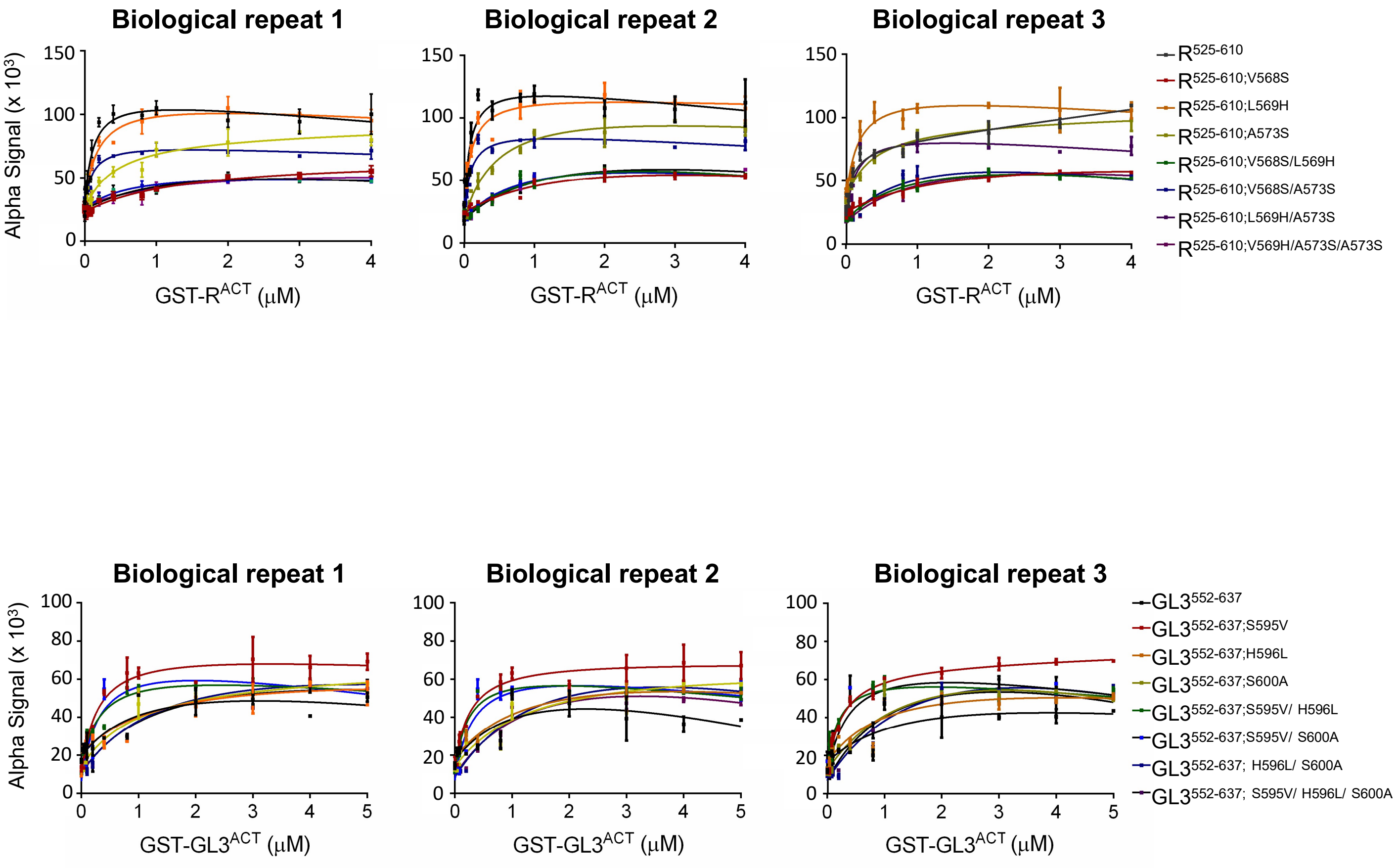

Figure S7. Homodimer binding affinities of R^{ACT}, $GL3^{ACT}$ and their respective mutants.

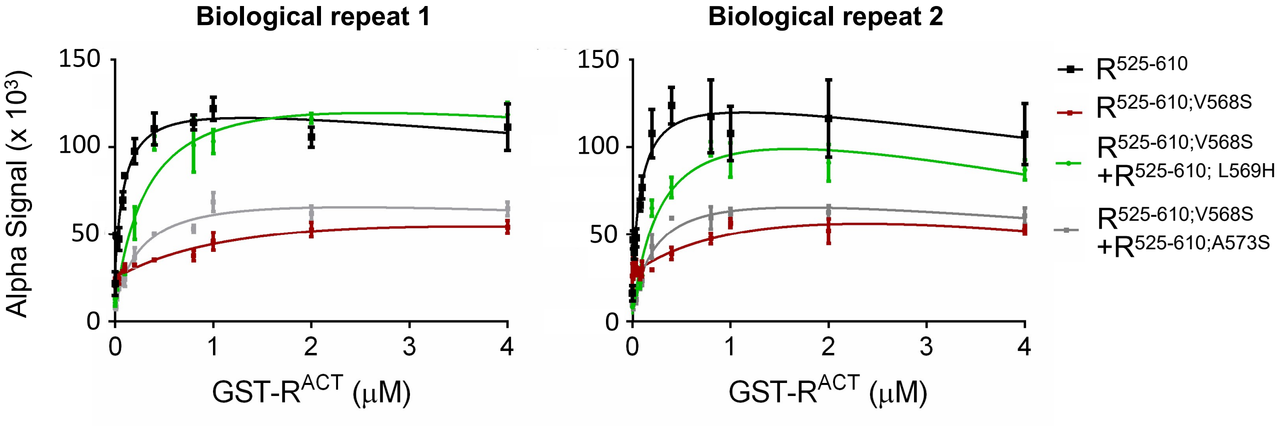

		$R^{525\text{-}610}$	$R^{525\text{-}610;V568S}$	$R^{525\text{-}610;V568S}$ $+R^{525\text{-}610;L569H}$	$R^{525\text{-}610;V568S}$ $+R^{525\text{-}610;A573S}$
Biological repeat 1	K_D (μM)	0.10	1.85	0.34	0.29
Biological repeat 2	K_D (μM)	0.09	1.78	0.31	0.25

Figure S8. Heterodimer binding affinities of R^{ACT} mutants.

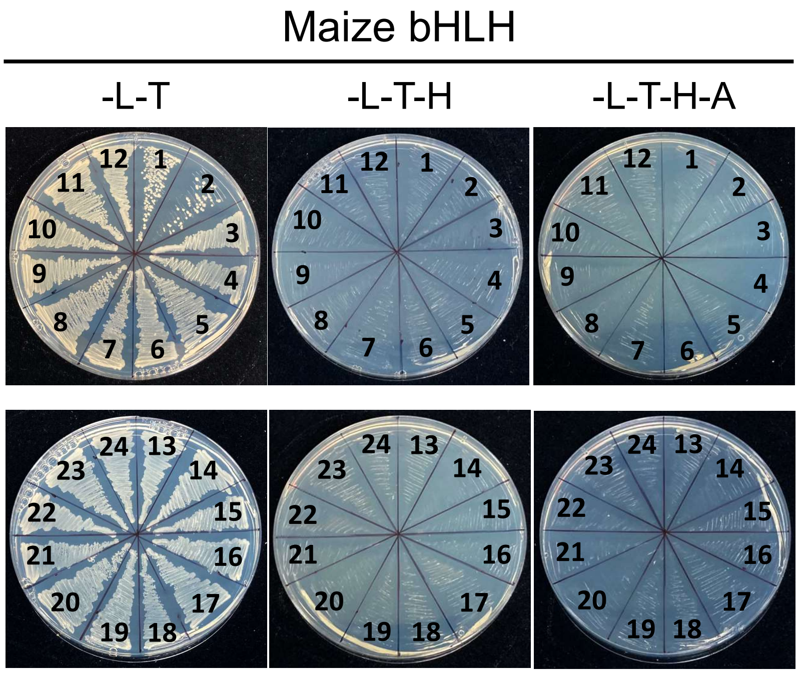

1.pAD-R$^{525-610}$	+pBD
2.pAD	+pBD-R$^{525-610}$
3.pAD-bHLH116$^{162-258}$	+pBD
4.pAD	+pBD-bHLH116$^{162-258}$
5.pAD-bHLH109$^{208-290}$	+pBD
6.pAD	+pBD-bHLH109$^{208-290}$
7.pAD-bHLH95$^{203-291}$	+pBD
8.pAD	+pBD-bHLH95$^{203-291}$
9.pAD-bHLH46$^{533-615}$	+pBD
10.pAD	+pBD-bHLH46$^{533-615}$
11.pAD-bHLH145$^{170-254}$	+pBD
12.pAD	+pBD-bHLH145$^{170-254}$
13.pAD-bHLH8$^{189-271}$	+pBD
14.pAD	+pBD-bHLH8$^{189-271}$
15.pAD-bHLH2$^{476-556}$	+pBD
16.pAD	+pBD-bHLH2$^{476-556}$
17.pAD-bHLH71$^{278-360}$	+pBD
18.pAD	+pBD-bHLH71$^{278-360}$
19.pAD-bHLH115$^{301-393}$	+pBD
20.pAD	+pBD-bHLH115$^{301-393}$
21.pAD-bHLH114$^{292-379}$	+pBD
22.pAD	+pBD-bHLH114$^{292-379}$
23. pAD-bHLH76$^{141-229}$	+pBD
24.pAD	+pBD-bHLH76$^{141-229}$

Figure S9. Autoactivation test of maize ACT-like domains in yeast.

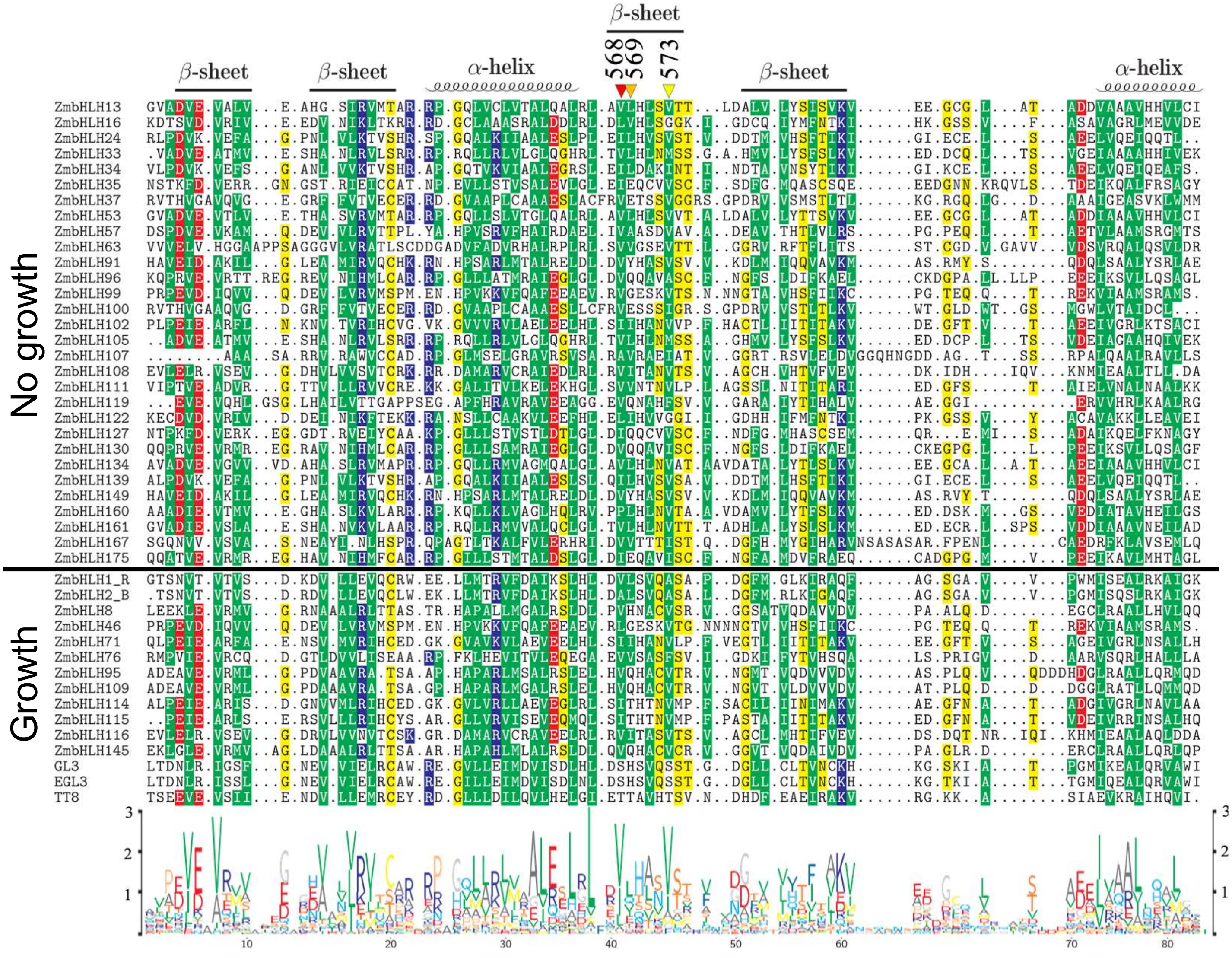

Figure S10. Sequence alignment of maize ACT-like domains.

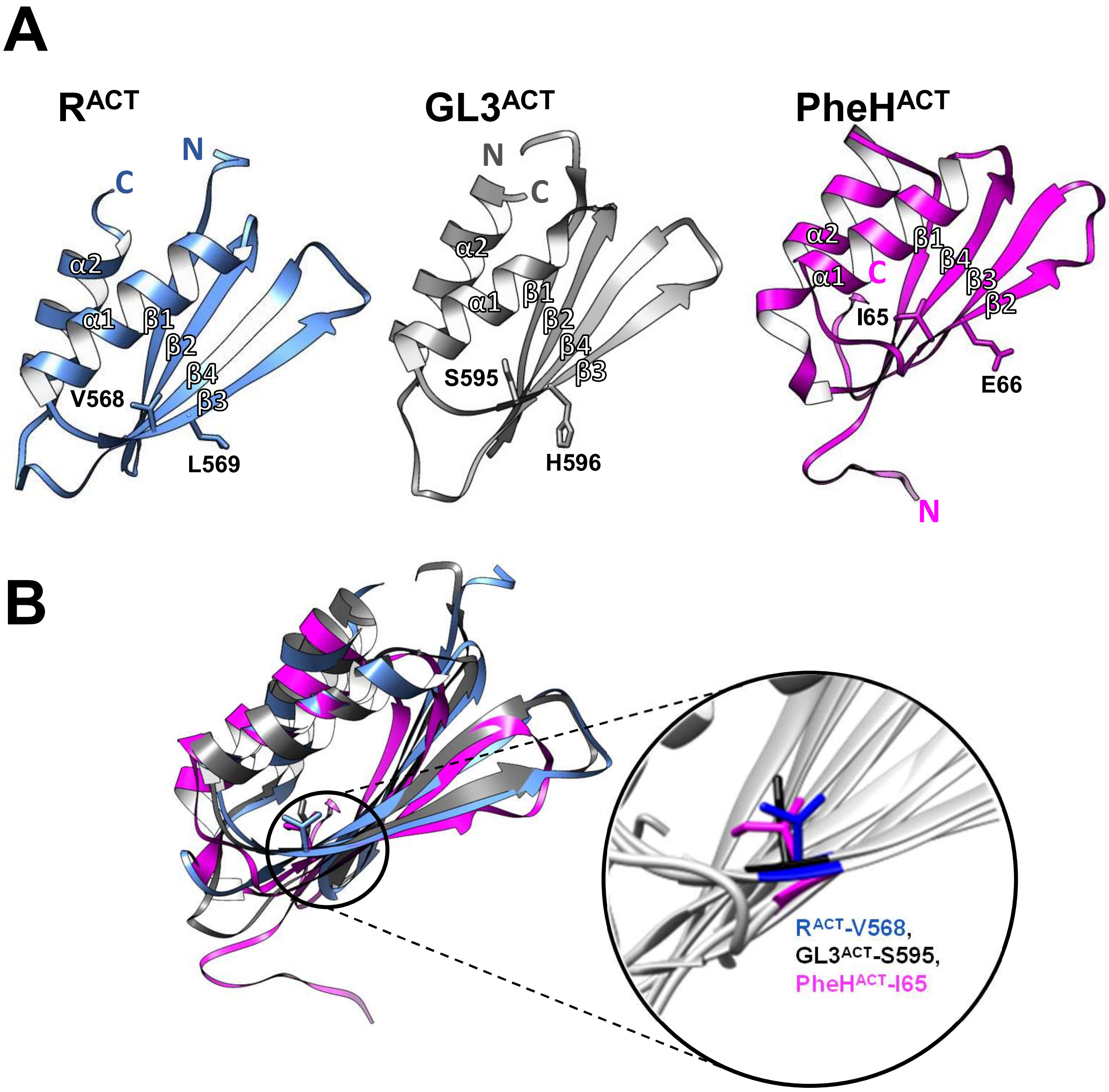

Figure S11. Comparison of predicted monomeric structures of R^{ACT}, GL3^{ACT}, and PheH^{ACT}

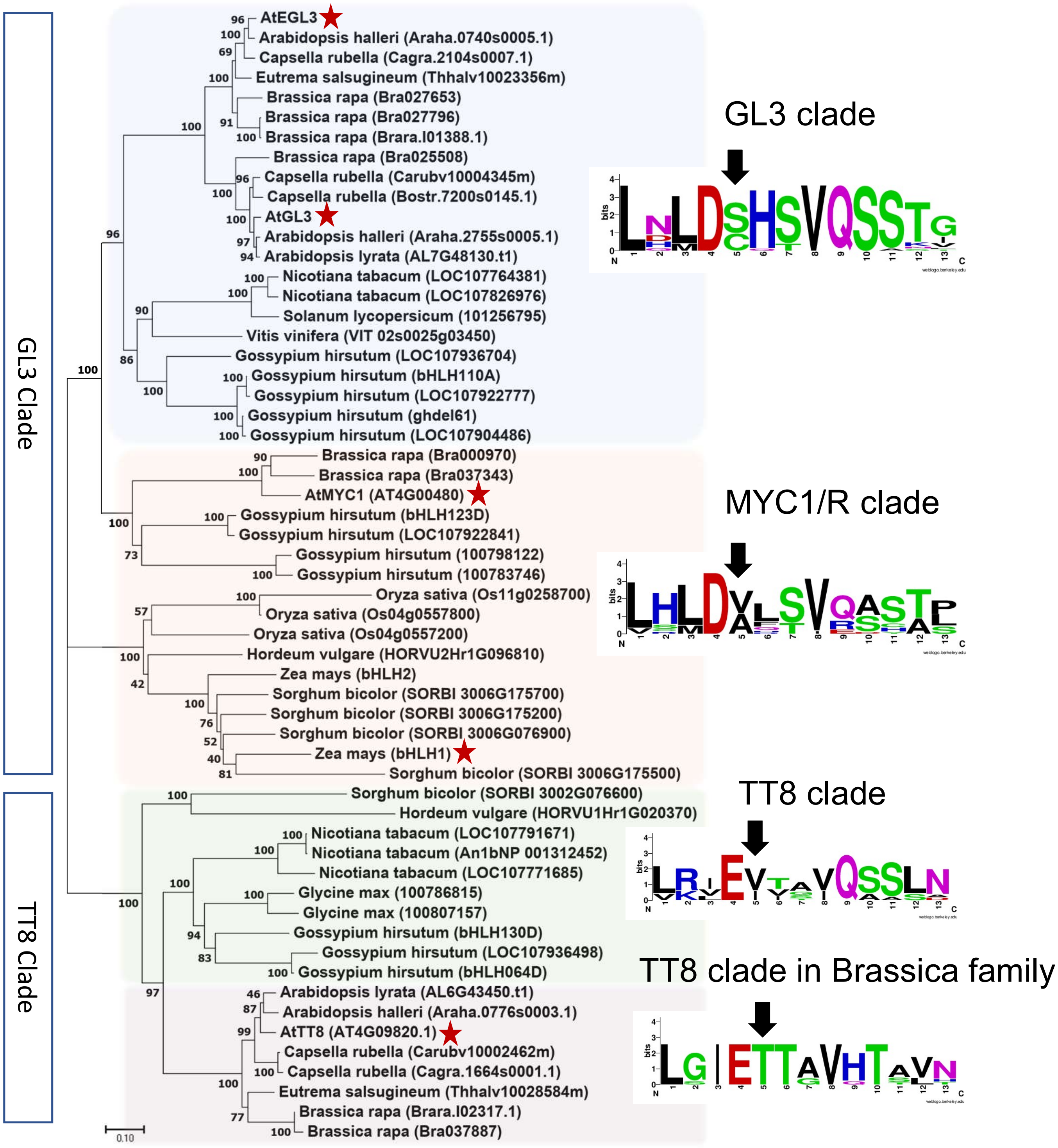

Figure S12. Distribution and sequence conservation of the predicted hydrophobic β-sheet among plant R orthologs.
